# Probing the Catalytic Mechanism and Inhibition of SAMHD1 Using the Differential Properties of R_p_- and S_p_-dNTPαS Diastereomers

**DOI:** 10.1021/acs.biochem.0c00944

**Published:** 2021-05-14

**Authors:** Elizabeth R. Morris, Simone Kunzelmann, Sarah J. Caswell, Andrew G. Purkiss, Geoff Kelly, Ian A. Taylor

**Affiliations:** † Macromolecular Structure Laboratory, The Francis Crick Institute, 1 Midland Road, London NW1 1AT, U.K.; ‡ Structural Biology Science Technology Platform, The Francis Crick Institute, 1 Midland Road, London NW1 1AT, U.K.; § The Medical Research Council Biomedical NMR Centre, The Francis Crick Institute, 1 Midland Road, London NW1 1AT, U.K.

## Abstract

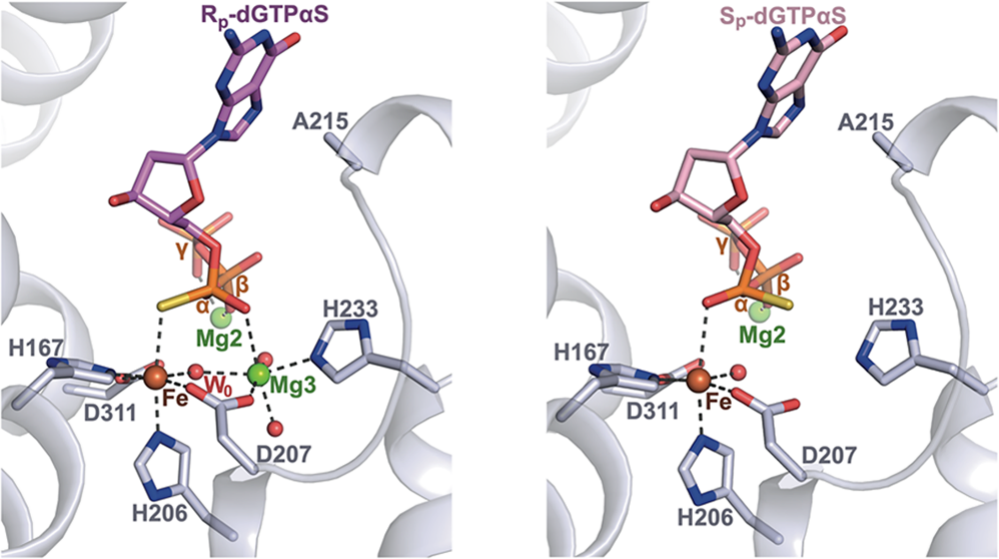

SAMHD1 is a fundamental regulator of cellular dNTPs that catalyzes their hydrolysis into 2′-deoxynucleoside and triphosphate, restricting the replication of viruses, including HIV-1, in CD4^+^ myeloid lineage and resting T-cells. SAMHD1 mutations are associated with the autoimmune disease Aicardi-Goutières syndrome (AGS) and certain cancers. More recently, SAMHD1 has been linked to anticancer drug resistance and the suppression of the interferon response to cytosolic nucleic acids after DNA damage. Here, we probe dNTP hydrolysis and inhibition of SAMHD1 using the R_p_ and S_p_ diastereomers of dNTPαS nucleotides. Our biochemical and enzymological data show that the α-phosphorothioate substitution in S_p_-dNTPαS but not R_p_-dNTPαS diastereomers prevents Mg^2+^ ion coordination at both the allosteric and catalytic sites, rendering SAMHD1 unable to form stable, catalytically active homotetramers or hydrolyze substrate dNTPs at the catalytic site. Furthermore, we find that S_p_-dNTPαS diastereomers competitively inhibit dNTP hydrolysis, while R_p_-dNTPαS nucleotides stabilize tetramerization and are hydrolyzed with similar kinetic parameters to cognate dNTPs. For the first time, we present a cocrystal structure of SAMHD1 with a substrate, R_p_-dGTPαS, in which an Fe–Mg-bridging water species is poised for nucleophilic attack on the P^α^. We conclude that it is the incompatibility of Mg^2+^, a hard Lewis acid, and the α-phosphorothioate thiol, a soft Lewis base, that prevents the S_p_-dNTPαS nucleotides coordinating in a catalytically productive conformation. On the basis of these data, we present a model for SAMHD1 stereospecific hydrolysis of R_p_-dNTPαS nucleotides and for a mode of competitive inhibition by S_p_-dNTPαS nucleotides that competes with formation of the enzyme–substrate complex.

Sterile alpha motif and HD domain containing protein 1 (SAMHD1) is a dNTP triphosphohydrolase enzyme that catalyzes the hydrolysis of dNTPs into triphosphate and 2′-deoxynucleoside.^1,2^ SAMHD1 is expressed in a variety of tissue types^3,4^ and is a key regulator of cellular dNTP homeostasis.^5^ In terminally differentiated myeloid lineage cells and resting T-cells, SAMHD1 activity reduces the dNTP pool to a level that inhibits the replication of HIV-1^6−8^ and other retroviruses^9^ as well as some DNA viruses.^10,11^ In addition to the restriction of viral infection, SAMHD1 is also an important effector of innate immunity, and SAMHD1 mutations are found in patients with the autoimmune disease AGS,^12^ early onset stroke,^13^ along with chronic leukemia^14,15^ and other cancers.^16−18^ High SAMHD1 expression in acute myeloid leukemia has been associated with reduced efficacy of the nucleoside analogue anticancer drugs Chlofarabine and Cytarabine,^19−21^ due to the hydrolysis of their active 5′-triphosphorylated forms by SAMHD1. More recently, SAMHD1 has been reported to have a triphosphohydrolase-independent function in genome maintenance pathways, facilitating homologous recombination^22^ and functioning in DNA repair to suppress the release of single-stranded DNA fragments from stalled replication forks into the cytosol.^23^

Human SAMHD1 is a 626-residue protein. It comprises an N-terminal nuclear localization signal,^24^ a sterile alpha motif (SAM) domain, and an HD phosphohydrolase domain^25^ containing the active site. In addition, C-terminal residues 600–626 are targeted by lentiviral Vpx accessory proteins to recruit SAMHD1 to the proteasome.^26,27^ The active form of SAMHD1 is a homotetramer^28^ where sequences N- and C-terminal to the HD domain stabilize intersubunit protein–protein interactions and incorporate four pairs of allosteric nucleotide-binding sites, AL1 and AL2, that regulate the enzyme through combined binding of G-based (AL1) and deoxynucleoside (AL2) triphosphates.^1,29−32^ The allosteric regulation of SAMHD1 has been studied extensively. Numerous studies have shown that GTP or dGTP are the physiological ligands for the first allosteric site, AL1,^31,33^ and that the second allosteric site, AL2, is specific for a dNTP with the following preference order: dATP > dGTP > TTP > dCTP.^34−37^ The AL1- and AL2-coordinated nucleotides are bridged by a single Mg^2+^ ion through their triphosphate moieties. SAMHD1 is also cell cycle regulated by cyclinA2/CDK2 phosphorylation at threonine 592^38−40^ through effects on tetramer stability that modulate activity,^33^ and removing this regulation may enable SAMHD1 to inhibit HIV-1 in cycling cells.^38^

The catalytic site of SAMHD1 can hydrolyze cognate dNTP substrates, with a preference for dCTP ≈ dGTP > TTP > dATP,^34^ as well as dNTP analogues such as 5′-triphosphorylated anticancer and antiviral agents.^41−43^ X-ray crystal structures of SAMHD1 in complex with substrate dNTPs and dNTP analogues have elucidated how SAMHD1 selectively binds these substrates^29,30,33−36,43^ and also utilizes the HD motif to tightly bind a Fe metal ion.^33,44^ Recently, we reported structures of SAMHD1 in complex with α,β-imido-dNTP (dNMPNPP) inhibitors, which enabled us to propose a mechanism for SAMHD1 dNTP hydrolysis involving a bimetallic Fe–Mg center that is shared by some HD domain enzymes.^44^ Modulation of SAMHD1 activity, for example, through inhibition by dNMPNPP nucleotide analogues, has been proposed as a therapeutic strategy for improving anticancer and antiviral therapeutic efficacy.^19,45,46^

We have now probed SAMHD1 catalysis and inhibition mechanisms using 2′-deoxynucleoside-5′-*O*-(1-thiotriphosphates) (dNTPαS) nucleotide analogues. Here, a nonbridging oxygen is replaced by sulfur at the α-phosphate of the dNTP, introducing a chiral center at the α-phosphorus (P^α^) and resulting in two diastereomers (R_p_-dNTPαS and S_p_-dNTPαS). Our enzymological data reveal that S_p_-dNTPαS diastereomers only weakly support SAMHD1 tetramerization, due to the hard/soft mismatch between the P^α^ phosphorothioate and the hard Lewis acid AL1-AL2-bridging Mg^2+^ that is required for tetramer assembly. We also determined that S_p_-dNTPαS nucleotides are competitive inhibitors of SAMHD1 catalysis with equilibrium inhibition constants, *K*_*i*_, in the micromolar range, as they bind in the active site but cannot maintain the metal and water ion coordination required to support nucleophilic attack on a substrate dNTP P^α^. By contrast, R_p_-dNTPαS nucleotides are SAMHD1 allosteric activators as well as substrates with kinetic parameters comparable with natural dNTP substrates. We cocrystallized R_p_-dGTPαS in AL1, AL2, and the active site of a catalytically inactive SAMHD1 mutant H215A, for the first time trapping a substrate in the active site with an Fe–Mg-bridging water species in line with the P^α^–O^5′^ scissile bond. On the basis of these data, we present a model for hydrolysis of R_p_-dNTPαS that supports a SAMHD1 catalytic mechanism that utilizes a bimetallic center and activated water molecule to hydrolyze dNTP substrates and describe a mode of inhibition by S_p_-dNTPαS nucleotides that competes with substrate dNTPs and prevents formation of an ES complex.

## Materials and Methods

### Protein Expression and Purification

For expression in *Escherichia coli*, the DNA sequences coding for human SAMHD1 residues M1–M626, SAMHD1 and Q109–M626, SAMHD1(109–626) were amplified by PCR and inserted into a pET52b expression vector (Novagen) using ligation-independent cloning (SAMHD1) or the *Xma*I/NotI restriction sites (SAMHD1(109–626) to produce N-terminal StrepII-tag fusion proteins. The H215A active site mutant was prepared from the parent Q109–M626 construct using the Quikchange II kit. Primer sequences for PCR and mutagenesis are provided in Table S2, and all insert sequences were verified by DNA sequencing. Strep-tagged SAMHD1 constructs were expressed in the *E. coli* strain Rosetta 2 (DE3) (Novagen) grown at 37 °C with shaking. Protein expression was induced by addition of 0.1 mM IPTG to log phase cultures (*A*_600_ = 0.5), and the cells were incubated for a further 20 h at 18 °C. Cells were harvested by centrifugation resuspended in 50 mL of lysis buffer (50 mM Tris-HCl pH 7.8, 500 mM NaCl, 4 mM MgCl_2_, 0.5 mM TCEP, 1× EDTA-free mini complete protease inhibitors (Roche), 0.1 U/mL benzonase (Novagen)) per 10 g of cell pellet and lysed by sonication. The lysate was cleared by centrifugation for 1 h at 50000*g* and 4 °C then applied to a 10 mL StrepTactin affinity column (IBA) followed by 300 mL of wash buffer (50 mM Tris-HCl pH 7.8, 500 mM NaCl, 4 mM MgCl_2_, 0.5 mM TCEP) at 4 °C. Bound proteins were eluted from the column by circulation of 0.5 mg of 3C protease (GE) in 25 mL of wash buffer over the column in a closed circuit overnight. 3C protease was removed by affinity chromatography using a 1 mL GSTrap column (GE), and the eluent was applied to a Superdex 200 26/60 (GE) size exclusion column equilibrated with 10 mM Tris-HCl pH 7.8, 150 mM NaCl, 4 mM MgCl_2_, 0.5 mM TCEP. Peak fractions were concentrated to approximately 20 mg mL^–1^ and flash frozen in liquid nitrogen in small aliquots.

### Nucleotides

Deoxyribonucleotide triphosphates and racemic mixtures of R_p_- and S_p_-dNTPαS nucleotides were purchased from Jena Biosciences Germany, DE, or TriLink Biotechnologies, US. Purified R_p_-dNTPαS and S_p_-dNTPαS diastereomers were from BioLog, DE.

### Crystallization and Structure Determination

Prior to crystallization, H215A-SAMHD1(109–626) was diluted to 5 mg mL^–1^ with gel filtration buffer, supplemented with 2 mM R_p_-dGTPαS. Crystals of the H215A-SAMHD1(109–626)-R_p_-dGTPαS–Mg complex were produced by sitting drop vapor diffusion at 18 °C using a mosquito crystal robot (SPT Labtech) to prepare 0.2 μL droplets containing an equal volume of the protein/nucleotide solution and mother liquor. The best crystals were obtained using a mother liquor of 0.1 M Bis-tris-HCl pH 6, 15% (w/v) PEG 3350, 0.15 M lithium sulfate. For data collection, the crystals were cryoprotected in mother liquor containing 30% (v/v) glycerol and flash frozen in liquid nitrogen. Data sets were collected on beamline I04 at the Diamond Light Source, UK, at a wavelength of 0.97949 Å. Details of the data collection, processing, and structure refinement statistics are presented in Table S1. Data were processed using the autoPROC pipeline^47^ (Global Phasing LtD). Internally, indexing and integration utilized XDS;^48,49^ point-group symmetry was determined with POINTLESS;^50^ isotropic scaling was carried out using AIMLESS;^51^ data were anisotropically scaled in autoPROC using STARANISO (http://staraniso.globalphasing.org/cgi-bin/staraniso.cgi) (Global Phasing LtD); and structure factors were generating using CTRUNCATE.^52^ The crystal belonged to the *P*2_1_2_1_2_1_ spacegroup with 8 copies of the H215A-SAMHD1(109–626) monomer and 24 copies of R_p_-dGTPαS in the asymmetric unit. The structure was solved by molecular replacement using the program PHASER^53^ implemented in the CCP4 interface^54^ employing the structure of H215A-SAMHD1(109–626) as search model (PDB code 6XU1(44)). Buccaneer^55^ and manual building within the program Coot^56^ were combined iteratively with refinement using individual B-factors and TLS refinement in Refmac5^57^ to produce a final model covering SAMHD1 residues 113–588 with *R*/*R*_free_-factors of 21.1/24.0%. The program AceDRG^58^ was used to derive the stereochemical restraint library for the nucleotide analogue R_p_-dGTPαS. In the model, 97.1% of residues have backbone dihedral angles in the favored region of the Ramachandran plot, a further 2.8% are in the allowed regions, and 0.1% are outliers. A simulated annealing composite omit map was generated using phenix.maps within the Phenix software package.^59^ The coordinates and structure factors of the H215A-SAMHD1(109–626)-R_p_-GTPαS complex have been deposited in the Protein Data Bank under accession number 7A5Y.

### SEC-MALLS

Size exclusion chromatography coupled to multi-angle laser light scattering (SEC-MALLS) was used to determine the molar mass composition of SAMHD1 samples upon addition of R_p_- and S_p_-dNTPαS nucleotide analogues and/or activators. SAMHD1 was incubated at 4 °C for 5 min after the addition of nucleotide analogues (0.5 mM) and activator (0.2 mM GTP), and then samples (100 μL) were applied to a Superdex 200 10/300 INCREASE GL column equilibrated in 20 mM Tris-HCl, 150 mM NaCl, 5 mM MgCl_2_, 0.5 mM TCEP, and 3 mM NaN_3_, pH 8.0, at a flow rate of 1.0 mL/min. The scattered light intensity and protein concentration of the column eluate were recorded using a DAWN-HELEOS laser photometer and an OPTILAB-rEX differential refractometer (dRI) (d*n*/d*c* = 0.186) respectively. The weight-averaged molecular mass of material contained in chromatographic peaks was determined using the combined data from both detectors in the ASTRA software version 6.1 (Wyatt Technology Corp., Santa Barbara, CA).

### NMR Analysis of SAMHD1 Catalysis

One-dimensional ^1^H NMR spectroscopy was used to measure SAMHD1 hydrolysis rates of dNTPs and R_p_- and S_p_-dNTPαS analogues. Reactions were prepared in NMR buffer (20 mM Tris-HCl pH 8.0, 150 mM NaCl, 5 mM MgCl_2_, 2 mM TCEP, 5% D_2_O) containing 0.5 mM of each dNTP or dNTPαS analogue, 100 μM GTP and 1 μM SAMHD1. In inhibition studies, 10–100 μM ZnCl_2_ or CdCl_2_ was additionally included in assays. ^1^H NMR spectra (two dummy scans, four scans) were recorded at 30 s intervals at 22 °C as a pseudo 2D array using a Bruker Avance 600 MHz NMR spectrometer equipped with a 5 mm TCI cryoprobe. Solvent suppression was achieved using excitation sculpting.^60^ Experiments were typically carried out for between 1 and 10 h. The integrals for clearly resolved substrate and product peaks at each time point were extracted using the Bruker Dynamics Centre software package and used to construct plots of substrate or product against time. Initial rates were extracted from the linear part of the curve in order to determine *k*_cat_ values. Under these conditions, the limit of detection is ∼5% product over the span of the experiment. This equates to a minimum detectable SAMHD1 normalized hydrolysis rate of 0.00075 s^–1^.

### Real-Time Measurement of Triphosphohydrolase Activity

To obtain quantitative kinetic parameters for substrate hydrolysis (*K*_M_ and *k*_cat_), SAMHD1 divalent metal ion dependencies and inhibition by S_p_-dNTPαS analogues (*K*_*i*_), a real-time continuous coupled assay was employed utilizing the biosensor MDCC-PBP^61,62^ to measure phosphate release from combined SAMHD1 triphosphohydrolase and *Saccharomyces cerevisiae* Ppx1 exopolyphosphatase activity.^42^ In a typical experiment, solutions containing wt-SAMHD1(1–626), Ppx1, MDCC-PBP, and GTP were incubated for 5 min in assay buffer (20 mM Tris pH 8.0, 150 mM NaCl, 5 mM MgCl_2_, and 2 mM TCEP) at 25 °C before the reaction was initiated by the addition of substrate nucleotides and nucleotide analogues. The final concentrations were 0.2 μM SAMHD1, 0.02 μM Ppx1, 40 μM MDCC-PBP, 0.2 mM GTP, and varying concentrations of dNTP substrates and dNTPαS analogues. In divalent metal ion titration experiments, an assay buffer without 5 mM MgCl_2_ was employed, and the different metal chloride salts MgCl_2_, MnCl_2_, CoCl_2_, NiCl_2_, ZnCl_2_, and CdCl_2_ were added over a concentration range of 0.1–10 mM. Throughout reactions the fluorescence intensity was recorded at 430 nm excitation and 465 nm emission wavelengths at 15–20 s time intervals over a period of 10–30 min in a Clariostar multiwell plate reader (BMG Labtech). Steady-state rates were obtained from time courses of P_i_ formation by linear regression of the data points in the linear phase of the reaction (<10% substrate consumed). The lower limit of detection under these conditions is ∼0.5 μM product accumulated over 20 min corresponding to a rate of 0.002 s^–1^). Rates were normalized for SAMHD1 concentration and plotted against substrate concentration. Michaelis constants (*K*_M_) and catalytic constants (*k*_cat_) for substrates were then determined by nonlinear least-squares fitting using either a Michaelis–Menten or Hill-function in the software package Prism 9 (Graphpad).

For inhibition studies, experiments were conducted at three constant substrate concentrations (1, 0.3, and 0.1 or 0.3, 0.1, and 0.03 mM TTP), the SAMHD1, Ppx1, MDCC-PBP and GTP concentrations were maintained as above, and the S_p_-dNTPαS inhibitor concentration was varied. The data from the three independent experiments were analyzed globally by nonlinear least-squares fitting using the equation for competitive inhibition (1); where *V*/[SAMHD1] is the steady-state rate, normalized to the SAMHD1 concentration, [S] is the (fixed) substrate concentration, [I] is the (variable) inhibitor concentration, *K*_*i*_ is the inhibition constant, and *k*_cat_ and *K*_M_ are the catalytic and Michaelis–Menten constants for substrate turnover in the absence of inhibitor.
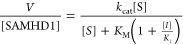
1The fitting was performed with a fixed value of *K*_M_ for GTP-activated TTP hydrolysis, determined previously,^42^ and only *k*_cat_ and *K*_*i*_ were allowed to vary. All measurements were performed in at least triplicate.

### RP-HPLC Analysis of R_p_-TTPαS and S_p_-TTPαS Hydrolysis

SAMHD1 rates of hydrolysis of equimolar mixtures of R_p_-TTPαS and S_p_-TTPαS were determined by reverse-phase chromatography analysis of reactions. Typically, 2 μM SAMHD1 was incubated with 0.2 mM GTP and 0.5 mM each of R_p_-TTPαS and S_p_-TTPαS in a reaction buffer of 20 mM Tris-HCl, 150 mM NaCl, 2 mM TCEP (pH 8.0) supplemented with either 5 mM MgCl_2_, 1 mM MnCl_2_, or 1 mM CoCl_2_. Samples were withdrawn at intervals from 0 to 60 min and quenched by 7-fold dilution into RP buffer (100 mM K_2_HPO_4_/KH_2_PO_4_ pH 6.5, 10 mM tetrabutylammonium bromide, 17% acetonitrile). R_p_-TTPαS, S_p_-TTPαS, and reaction products were then separated from precipitated protein by filtration through a 0.22 μm centrifugal filter (Durapore-PVDF, Millipore). Samples (5 nmol) were applied to a Zorbax SB-C18 column (4.6 × 250 mm, 3.5 μm, 80 Å pore size, Agilent Technologies), maintained at 30 °C, and mounted on a Jasco HPLC system controlled by Chromnav software (v1.19, Jasco). The thymidine reaction product (*R*_t_ = 2.5 min), activator GTP (*R*_t_ = 3.9 min), and substrates (R_p_-TTPαS (R_t_ = 8.6 min) and S_p_-TTPαS (*R*_t_ = 7.7 min) were separated under isocratic flow by application of RP buffer at 1 mL min^–1^ over 15 min. Absorbance data from the column eluent were continuously monitored between 200 and 650 nm (1 nm intervals) using an MD-2010 photodiode array detector (Jasco). The amount of R_p_-TTPαS and S_p_-TTPαS throughout the course of the reaction was determined by peak integration of the 260 nm absorbance data. Rates were determined by linear regression of a plot of the amount of R_p_-TTPαS and S_p_-TTPαS against reaction time.

## Results

### R_p_- and S_p_-dNTPαS Diastereomers

**Figure 1:**
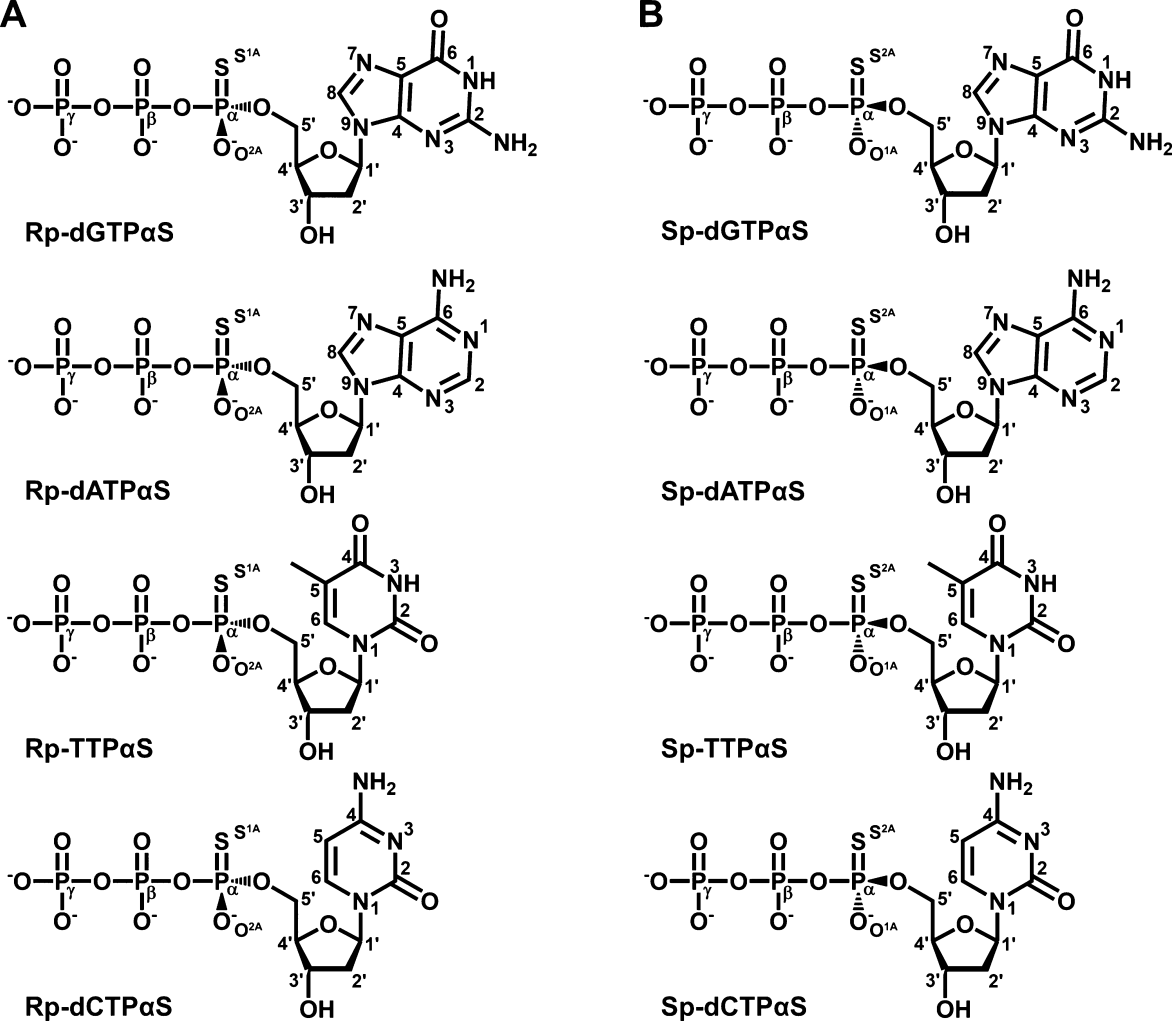
Chemical structures of deoxynucleotide analogues. Diagrammatic representations of the chemical structures of the (A) R_p_-dNTPαS and (B) S_p_-dNTPαS analogues employed in this study. Base and sugar carbon and nitrogen atoms are numbered using the standard convention for purine- and pyrimidine-based nucleotides. The α-phosphate nonbridging sulfur and oxygen are labeled using the nomenclature from ref (63).

The substitution by sulfur of a nonbridging diastereotopic oxygen at the α-phosphate of a dNTP introduces a chiral center at P^α^, with the replacement of the *pro-R* and *pro-S* oxygen atoms resulting in the formation of the R_p_-dNTPαS and S_p_-dNTPαS diastereomers containing S^1A^–O^2A^ and O^1A^–S^2A^ atoms respectively^63^ (Figure 1). Given that the incorporation of R_p_ and S_p_ diastereomers into nucleotides and nucleic acids often results in differential properties with respect to the action of stereoselective enzymes and receptors,^64−68^ we sought to test the ability of R_p_-dNTPαS and S_p_-dNTPαS analogues to support SAMHD1 tetramerization through binding at AL1 and AL2 and assess their properties as substrates or inhibitors at the SAMHD1 active site.

### SAMHD1 Allosteric Sites Are Selective for R_p_- over S_p_-dNTPαS

**Figure 2:**
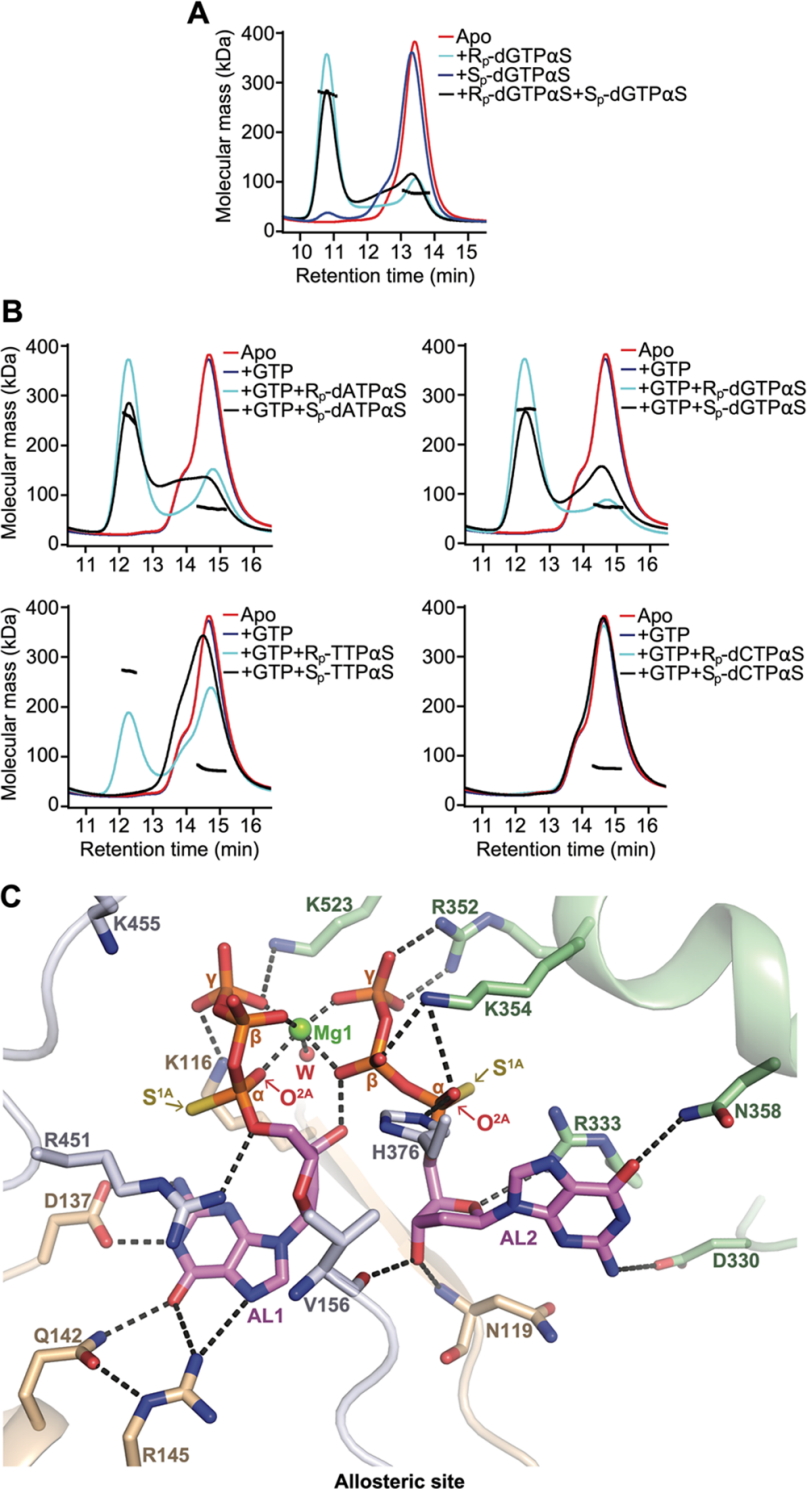
SAMHD1 tetramerization of R_p_-dNTPαS and S_p_-dNTPαS nucleotides. (A) SEC-MALLS analysis of SAMHD1 monomer–dimer-tetramer equilibrium upon addition of R_p_-dGTPαS and S_p_-dGTPαS nucleotides. The solid lines are the chromatograms from the output of the differential refractometer, and the black scatter points are the weight-averaged molar masses determined at 1-s intervals throughout elution of chromatographic peaks, SAMHD1 monomer–dimers elute at 12.5–14.5 min, tetramers at 11 min. The displayed chromatograms are apo-SAMHD1 (red); SAMHD1 and 0.5 mM R_p_-dGTPαS (cyan); SAMHD1 and 0.5 mM S_p_-dGTPαS (blue); SAMHD1 and 0.5 mM R_p_-dGTPαS + S_p_-dGTPαS (black). (B) SEC-MALLS analysis of SAMHD1 monomer–dimer–tetramer equilibrium upon addition of R_p_-dNTPαS or S_p_-dNTPαS nucleotides and GTP. SAMHD1 monomer–dimers elute at 14–16 min, tetramers at 12.5 min. Chromatograms are apo-SAMHD1 (red); SAMHD1 and 0.2 mM GTP (blue); SAMHD1, 0.2 mM GTP and 0.5 mM indicated R_p_-dNTPαS analogue (cyan); SAMHD1, 0.2 mM GTP and 0.5 mM indicated S_p_-dNTPαS analogue (black). (C) View of the allosteric site in the H215A-SAMHD1(109–626)-R_p_-dGTPαS structure. The protein backbone is shown in cartoon representation, bound R_p_-dGTPαS nucleotides are shown in stick representation in violet, and the coordinated Mg ion (Mg1) and water molecule are shown as spheres. Residues that make interactions with the nucleotides are labeled, and hydrogen bonding and coordinate bonds are shown as dashed lines. The S^1A^ sulfur and the O^2A^ oxygen atoms that make hydrogen bonding interactions with the AL1- and AL2-bound nucleotides are indicated. The configuration of these oxygen and sulfur atoms would be exchanged in S_p_-dNTPαS nucleotides and would disrupt the hydrogen bonding network of the allosteric site.

We first analyzed the ability of R_p_- and S_p_-dNTPαS diastereomers to support SAMHD1 tetramerization through binding at allosteric sites AL1 and AL2, which is required for catalysis. SEC-MALLS analysis of SAMHD1 tetramerization showed that in the absence of GTP, R_p_-dGTPαS strongly induced SAMHD1 tetramerization, S_p_-dGTPαS was ineffectual, but an equimolar mixture of R_p_-dGTPαS and S_p_-dGTPαS induced a similar level of tetramerization as R_p_-dGTPαS alone (Figure 2A).

These data demonstrate that R_p_-dGTPαS is sufficient to bind at both AL1 and AL2 to induce tetramer formation, while S_p_-dGTPαS is impaired in binding either at AL1 or AL2 or both. Further SEC-MALLS data that included GTP (Figure 2B) show that the R_p_-diastereomers of dGTPαS, dATPαS, and TTPαS generally stabilized SAMHD1 tetramerization, through AL2-binding, more than their S_p_-diastereomer counterparts, and dCTPαS diastereomers did not induce significant tetramerization, as previously reported for dCTP^35^ as well as dCMPNPP and α,β-methyleno-dCTP (dCMPCPP) analogues.^44^ Therefore, these data demonstrate that there is a clear preference for R_p_- over S_p_-dGTPαS in AL1 and for R_p_- over S_p_-dNTPαS nucleotides in AL2.

To further investigate this preference, we cocrystallized the catalytic domain, residues 109–626, of a catalytically inactive H215A SAMHD1 mutant^44^ with R_p_-dGTPαS and magnesium ions. The structure of this H215A-SAMHD1(109–626)-R_p_-dGTPαS–Mg complex was determined by molecular replacement to 2.3 Å resolution and contains two SAMHD1 tetramers in the asymmetric unit (Figure S1) with electron density for nucleotides and metal ions found in each of the allosteric and active sites (Figures S2 and S3). Details of the data collection and structure refinement are presented in Table S1. Inspection of the allosteric site of this H215-SAMHD1(109–626)-R_p_-dGTPαS–Mg complex (Figure 2C) provides a structural explanation for the observed preference for R_p_- over S_p_-dNTPαS nucleotides. Here, it is apparent that AL1 selectivity for R_p_-dGTPαS results from the α-phosphate O^2A^ oxygen of the AL1-bound nucleotide that coordinates the AL1-AL2-bridging magnesium ion (Mg1). This interaction would be disrupted by the *pro-S* thio-substitution in S_p_-dGTPαS due to the incompatibility of a hard Lewis acid (Mg1) and a soft Lewis base (P^α^-phosphorothioate). In addition, AL2 selectivity for R_p_-dNTPαS diastereomers results from hydrogen bonding between Lys354 (N^ζ^H), His376 (N^ε2^H), and the O^2A^ oxygen in the AL2-coordinated nucleotide, which would again be perturbed by the thiol substitution in S_p_-dNTPαS nucleotides. These observations are further supported by a previous study where SAMHD1 was cocrystallized with a R_p_- and S_p_-dGTPαS racemic mixture.^29^ There, only R_p_-dGTPαS was observed in the allosteric site,^29^ suggesting a strong selectivity preference for the R_p_ over the S_p_ diastereomer.

### R_p_- but not S_p_-dNTPαS Are Hydrolyzed by GTP-Activated SAMHD1

**Figure 3:**
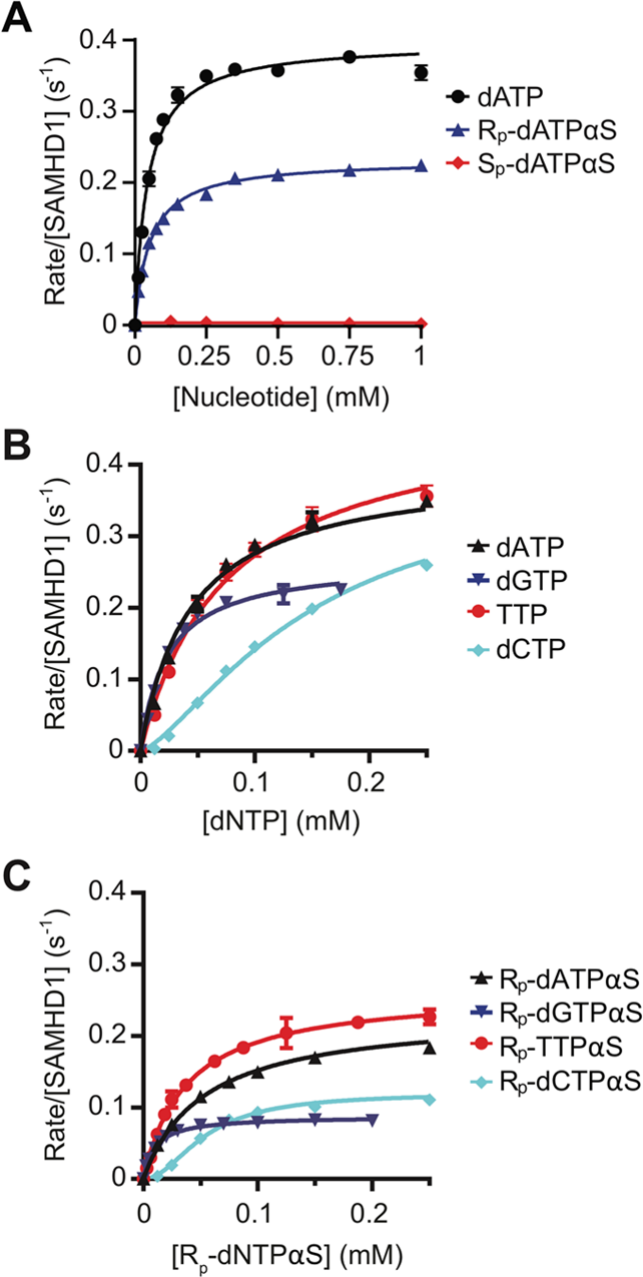
Steady-state kinetics of SAMHD1 hydrolysis of dNTPs and R_p_-dNTPαS and S_p_-dNTPαS analogues. (A) Steady-state kinetic analysis of GTP-stimulated hydrolysis of dATP, R_p_-dATPαS, and S_p_-dATPαS by SAMHD1. The dependence of the enzyme-normalized rate on substrate concentration are plotted, (black) dATP, (blue) R_p_-dATPαS, and (red) S_p_-dATPαS. For the dATP and R_p_-dATPαS reactions, the solid line is the best fit to the data using the Michaelis–Menten expression, which gives values for the derived constants *K*_M_ and *k*_cat_, of 44 ± 3 μM and 0.4 ± 0.01 s^–1^ for dATP and 53 ± 2 μM and 0.23 ± 0.01 s^–1^ for R_p_-dATPαS respectively. (B) Steady-state kinetic analysis of GTP-stimulated SAMHD1 hydrolysis of dNTPs. (C) Steady-state kinetic analysis of GTP-stimulated hydrolysis of R_p_-dNTPαS analogues by SAMHD1. In B and C, the dependence of the enzyme-normalized rate on substrate concentration is plotted in each panel (black) dATP, R_p_-dATPαS; (blue) dGTP, R_p_-dGTPαS; (red) TTP, R_p_-TTPαS, and (cyan) dCTP, R_p_-dCTPαS. The solid line is the best fit to the data using the Michaelis–Menten equation, or the Hill-modified equation for dCTP and R_p_-dCTPαS. Values for the derived constants *K*_M_ and *k*_cat_ from the data presented in A–C are listed in Table 1; error bars represent the standard error of the mean (SEM) of at least three independent measurements.

In order to inform the SAMHD1 dNTP hydrolysis mechanism, the properties of R_p_- and S_p_-dNTPαS nucleotides with respect to SAMHD1 catalytic activity were assessed using a fluorescence-based coupled enzyme assay^42^ and by ^1^H NMR spectroscopy. Data from the coupled enzyme assay revealed that R_p_-dATPαS was hydrolyzed with a similar Michaelis constant (*K*_M_) as dATP but with about a 2-fold reduction in catalytic rate constant (*k*_cat_) in a GTP-stimulated reaction (Figure 3A and Table 1). By contrast, no measurable hydrolysis of S_p_-dATPαS was observed above the limit of detection (<0.002 s^–1^) (Figure 3A). Examination of the hydrolysis of other R_p_-diastereomers (R_p_-dGTPαS, R_p_-TTPαS, and R_p_-dCTPαS) showed a 2–3 fold variation in *K*_M_ values relative to canonical nucleotides and 2–3 fold reductions in *k*_cat_ (Figure 3B,C and Table 1) but with the same rank order of turnover TTP > dATP > dCTP > dGTP. However, with both dCTP and R_p_-dCTPαS, significant sigmoidal behavior is apparent, likely as a result of poor AL2 binding, and so Hill coefficients (*h*) were applied to adequately fit the data (Table 1). Nevertheless, these data clearly demonstrate that in the presence of GTP all R_p_-dNTPαS nucleotides are hydrolyzed by SAMHD1 with kinetic constants comparable to the canonical nucleotides.

**Table 1:** SAMHD1 Catalytic Parameters for dNTP and R_p_-dNTPαS Nucleotides

substrate	AL1 activator	AL2 activator	*K*_M_ (μM)	*h*^a^	*k*_cat_ (s^–1^)
dATP	GTP	dATP	44 ± 3		0.40 ± 0.01^b^
dGTP	GTP/dGTP	dGTP	24 ± 2		0.27 ± 0.02
TTP	GTP	TTP	75 ± 6		0.48 ± 0.04
dCTP	GTP	dCTP	151 ± 6	1.4 ± 0.1	0.40 ± 0.01
R_p_-dATPαS	GTP	R_p_-dATPαS	53 ± 2		0.23 ± 0.01
R_p_-dGTPαS	GTP/R_p_-dGTPαS	R_p_-dGTPαS	10 ± 0.5		0.09 ± 0.01
R_p_-TTPαS	GTP	R_p_-TTPαS	38 ± 4		0.26 ± 0.01
R_p_-dCTPαS	GTP	R_p_-dCTPαS	54 ± 4	2.0 ± 0.2	0.12 ± 0.01

a For GTP/dCTP and GTP/R_p_-dCTPαS, *K*_M_ is derived from a Hill equation *V* = (*V*_max_[S]^*h*^)/(*K*_M_^*h*^ + [S]*^h^*) where *h* is the Hill coefficient for substrate binding;

b Error is the SEM of at least three independent measurements.

**Figure 4:**
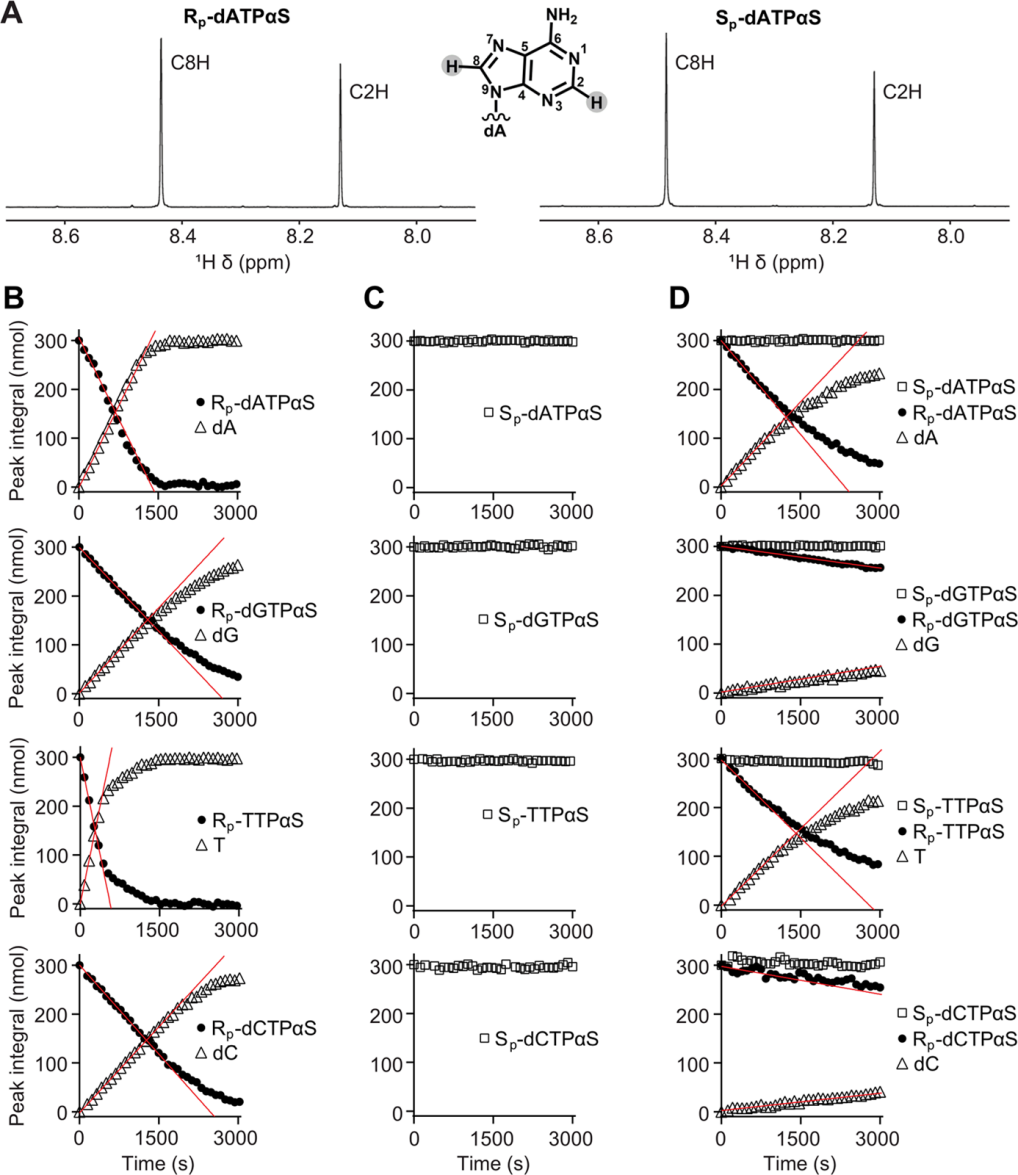
^1^H NMR analysis of SAMHD1 hydrolysis of R_p_-dNTPαS and S_p_-dNTPαS analogues. (A) Downfield nucleobase region of the ^1^H NMR spectra of R_p_-dATPαS (left) and S_p_-dATPαS (right) diastereomers. The two singlet peaks are the resonances from the C8H and C2H protons of the adenine base; R_p_-dATPαS chemical shifts are 8.431 and 8.140 ppm respectively; S_p_-dATPαS 8.463 and 8.145 ppm, respectively. Inset is the chemical structure of an adenine base, numbered according to standard convention. (B) ^1^H NMR analysis of GTP-activated R_p_-dNTPαShydrolysis. (C) ^1^H NMR analysis of GTP-activated S_p_-dNTPαShydrolysis. (D) ^1^H NMR analysis of GTP-activated, hydrolysis of an equimolar mixture of R_p_- and S_p_-dNTPαS diastereomers by SAMHD1. In B, C, and D, data were recorded for SAMHD1 hydrolysis reactions containing 1 μM SAMHD1, 0.2 mM GTP AL1-activator, and 0.5 mM R_p_-dNTPαS (filled circle, B), S_p_-dNTPαS (open square, C), or both (D). In each panel, the integral of resolved substrate and product (open triangle) peak resonances are plotted against time. Rates of hydrolysis were determined from slopes (red lines) derived from the data measured in the linear phase of the reaction, presented in Table 2 and Table 3. In C and D, no significant reduction in the S_p_-dNTPαS peak intensities is apparent, indicating that S_p_-dNTPαS analogues are refractory to SAMHD1 hydrolysis.

Hydrolysis of R_p_- and S_p_-dNTPαS nucleotides by SAMHD1 was also investigated using ^1^H NMR spectroscopy that readily distinguishes R_p_- and S_p_-dNTPαS diastereomers by their ^1^H NMR spectrum. The spectra of R_p_-dATPαS and S_p_-dATPαS (Figure 4A) contain two singlet peaks in the downfield nucleobase region from the C8H and C2H protons of the adenine base. The chemical shifts of the R_p_-dATPαS C8H and C2H protons are 8.431 and 8.140 ppm, and the S_p_-dATPαS C8H and C2H have chemical shifts of 8.463 and 8.145 ppm. Other dNTPαS diastereomers are also distinguishable by the unique resonances of base protons. Therefore, ^1^H NMR was used to measure GTP-stimulated SAMHD1 hydrolysis of each R_p_- and S_p_-dNTPαS diastereomer. These data (Figure 4B,C) clearly demonstrate that, while R_p_-dNTPαS diastereomers are SAMHD1 substrates, the S_p_-dNTPαS diastereomers are refractory to hydrolysis, in good agreement with observations from the coupled enzyme assay (Figure 3). Moreover, the apparent *k*_cat_ values measured for R_p_-dNTPαS substrates were 2–4 fold lower than those of the canonical dNTPs (Table 2) with a rank order of hydrolysis of R_p_-TTPαS > R_p_-dATPαS > R_p_-dGTPαS ≈ R_p_-dCTPαS, mirroring that of the canonical dNTPs (TTP > dATP > dGTP > dCTP) in a ^1^H NMR assay^44^ and close to that observed in the coupled enzyme assay (Table 1).

**Table 2:** SAMHD1 Catalytic Turnover of R_p_-dNTPαS Nucleotides

substrate	AL1 activator	AL2 activator	*k*_cat_ (s^–1^)
dATP	GTP	dATP	0.86 ± 0.09^a^^,^^b^
dGTP	GTP/dGTP	dGTP	0.66 ± 0.15^a^
TTP	GTP	TTP	1.43 ± 0.07^a^
dCTP	GTP	dCTP	0.57 ± 0.11^a^
R_p_-dATPαS	GTP	R_p_-dATPαS	0.33 ± 0.01
R_p_-dGTPαS	GTP/R_p_-dGTPαS	R_p_-dGTPαS	0.192 ± 0.001
R_p_-TTPαS	GTP	R_p_-TTPαS	0.885 ± 0.001
R_p_-dCTPαS	GTP	R_p_-dCTPαS	0.199 ± 0.004

a Values for hydrolysis of canonical dNTPs from ref (38).

b Error is the SEM of at least two independent measurements.

### S_p_-dNTPαS Diastereomers Inhibit SAMHD1 Catalysis

Having demonstrated that R_p_-dNTPαS diastereomers can stabilize SAMHD1 tetramers through AL2-binding and that they are hydrolyzed by SAMHD1 with catalytic parameters similar to their cognate canonical dNTP, we next wanted to investigate the refractory S_p_-dNTPαS diastereomers in the context of SAMHD1 catalysis. SEC-MALLS experiments showed that the stabilization of SAMHD1 tetramers through AL2 binding of S_p_-dNTPαS was much less than that by R_p_-dNTPαS (Figure 2B). Therefore, the lack of hydrolysis in ^1^H NMR and coupled enzyme fluorescence experiments (Figures 3 and 4B–C) may either be a result of using a poor AL2 activator or that S_p_-dNTPαS diastereomers are directly refractory to hydrolysis by the SAMHD1 active site. To test these ideas and promote tetramerization of SAMHD1 in ^1^H NMR assays measuring S_p_-dNTPαS hydrolysis, we combined GTP and a 1:1 mix of each R_p_- and S_p_-dNTPαS pair and simultaneously monitored both R_p_- and S_p_-dNTPαS as substrates (Figure 4D). Analysis of these experiments reveals three key observations. First, all R_p_-dNTPαS diastereomers are hydrolyzed, confirming tetramerization of SAMHD1 through AL2 binding. Second, all the S_p_-dNTPαS diastereomers are still refractory to hydrolysis, indicating that, although SAMHD1 is activated through AL2 binding by R_p_-dNTPαS, S_p_-dNTPαS diastereomers are not hydrolyzed at the active site. Third, although the R_p_-dNTPαS diastereomers are still hydrolyzed, they are hydrolyzed at significantly reduced rates, 2–8-fold slower than in the absence of S_p_-dNTPαS (Table 3). Thus, we concluded that not only are S_p_-dNTPαS diastereomers refractory to hydrolysis they are competitive inhibitors of SAMHD1 nucleotide hydrolysis through binding at the active site.

**Table 3:** S_p_-dNTPαS Inhibition of SAMHD1 R_p_-dNTPαS Hydrolysis

substrate	S_p_-dNTPαS	*k*_cat_ (s^–1^)	fold reduction^b^
R_p_-dATPαS		0.33 ± 0.01^a^	1.7
R_p_-dATPαS	S_p_-dATPαS	0.20 ± 0.01	
R_p_-dGTPαS		0.192 ± 0.001	7.1
R_p_-dGTPαS	S_p_-dGTPαS	0.027 ± 0.001	
R_p_-TTPαS		0.885 ± 0.001	4.9
R_p_-TTPαS	S_p_-TTPαS	0.181 ± 0.004	
R_p_-dCTPαS		0.199 ± 0.004	7.7
R_p_-dCTPαS	S_p_-dCTPαS	0.026 ± 0.006	

a Error is the SEM derived of at least two independent measurements.

b Fold reduction is the ratio of *k*_cat_ for hydrolysis of each R_p_-dNTPαS diastereomer in the absence or presence of the S_p_-dNTP diastereomer.

**Figure 5:**
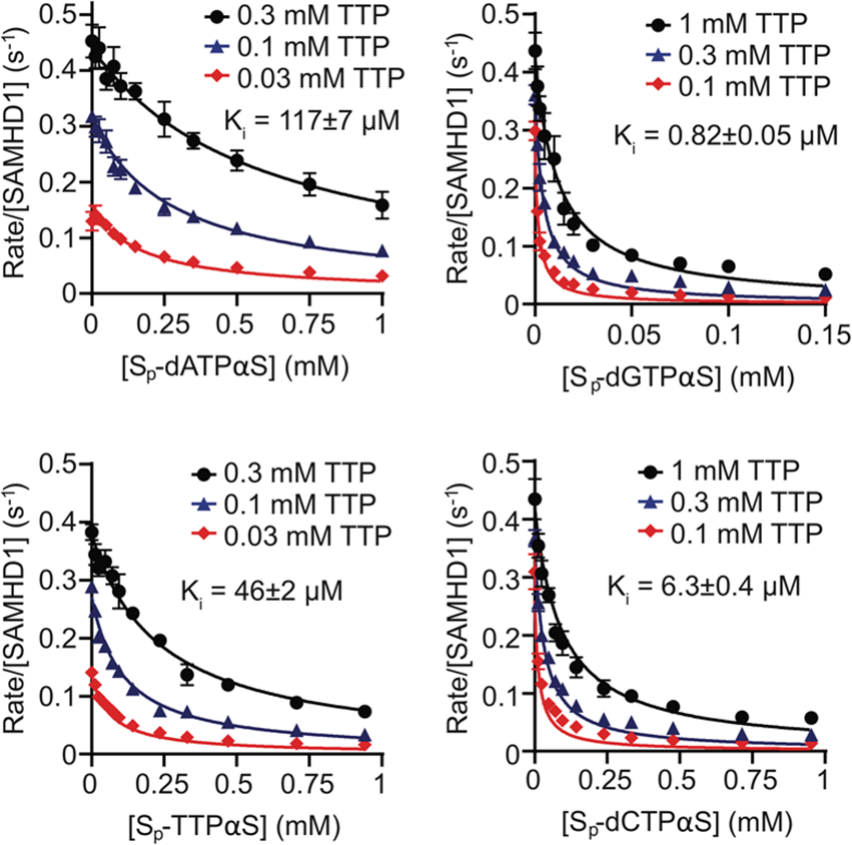
Inhibition of SAMHD1 hydrolysis by S_p_-dNTPαS deoxynucleotides. Determination of S_p_-dNTPαS inhibition constants (*K*_*i*_). Plots show the dependence of the SAMHD1 hydrolysis rate of 0.03, 0.1, and 0.3 mM TTP (S_p_-dATPαS and S_p_-TTPαS) or 0.1, 0.3, and 1 mM TTP (S_p_-dGTPαS and S_p_-dCTPαS) on the concentration of S_p_-dNTPαS nucleotides. The reported *K*_*i*_ values (inset and Table 4) were derived from global fitting of each three-concentration data set. Error bars represent the standard error of the mean (SEM) of at least three independent measurements.

These data provide semiquantitative measurements of competitive inhibition by S_p_-dNTPαS diastereomers. Therefore, further studies using enzyme-coupled inhibition assays were undertaken to determine the inhibition constant (*K*_*i*_) for each S_p_-dNTPαS for the GTP-activated hydrolysis of a TTP substrate by SAMHD1. These data fit well to a competitive inhibition model, demonstrating that all S_p_-dNTPαS diastereomers competitively inhibit SAMHD1 triphosphohydrolase activity (Figure 5) with *K*_*i*_ ranging from 117 μM for S_p_-dATPαS to 0.82 μM for S_p_-dGTPαS with a rank order of *K*_*i*_ of S_p_-dATPαS > S_p_-TTPαS > S_p_-dCTPαS > S_p_-dGTPαS (Table 4) that mirrors the same nucleobase rank order as observed previously with the dNMPNPP inhibitors.^44^

**Table 4:** S_p_-dNTPαS Inhibition of SAMHD1 TTP Hydrolysis

inhibitor	AL1 activator	substrate	*K*_*i*_ (μM)
S_p_-dATPαS	GTP	TTP	117 ± 7^a^
S_p_-dGTPαS	GTP	TTP	0.82 ± 0.05
S_p_-TTPαS	GTP	TTP	46 ± 2
S_p_-dCTPαS	GTP	TTP	6.3 ± 0.4

a Error is the SEM of at least three independent measurements.

### Conformation of R_p_- and S_p_-dNTPαS Diastereomers in the SAMHD1 Active Site

**Figure 6:**
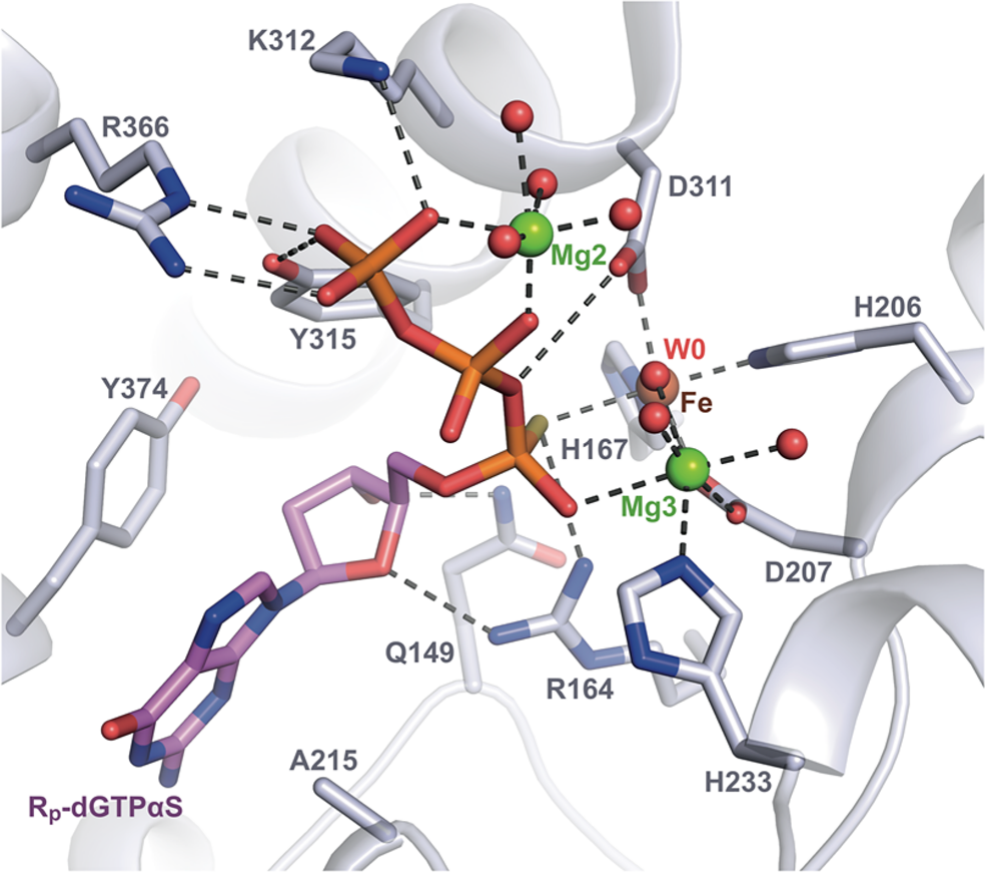
Residues that coordinate R_p_-dGTPαS in the H215A active site. The SAMHD1 protein backbone is shown in cartoon representation, in blue-white. The active site-bound R_p_-dGTPαS nucleotide and surrounding residues are shown in stick representation. Fe and Mg ions are represented as brown and green spheres, respectively. Coordinated waters are shown as red spheres.

The H215A-SAMHD1(109–626)-R_p_-dGTPαS crystal structure contains a R_p_-dGTPαS substrate bound at the active site (Figure 6 and Figure S3), as well as in allosteric sites AL1 and AL2. In this structure, the active site R_p_-dGTPαS coordinates the His/Asp-bound Fe, two Mg ions (Mg2 and Mg3), and hydrating water molecules. Several amino acids also interact with or pack against the guanine base, 2′-deoxyribose and thio-substituted triphosphate, including Gln149, Arg164, His210, Lys312, Tyr315, Arg366, and Tyr374. Although Ala215, that replaces histidine in the H215A mutant, cannot provide the general acid required for catalysis of the substrate R_p_-dGTPαS, an Fe/Mg3-bridged water, W0, that could act as a nucleophile for catalysis is positioned in line with the scissile P^α^–O^5′^ phosphoester bond of the substrate R_p_-dGTPαS (Figure 6). This suggests that the R_p_-dGTPαS substrate conformation in the active site is representative of the precatalytic state and is consistent with our enzymological data, which demonstrates that the R_p_-dNTPαS diastereomers are substrates, albeit with a small reduction in *k*_cat_ relative to canonical dNTPs (Figures 3 and 4).

**Figure 7:**
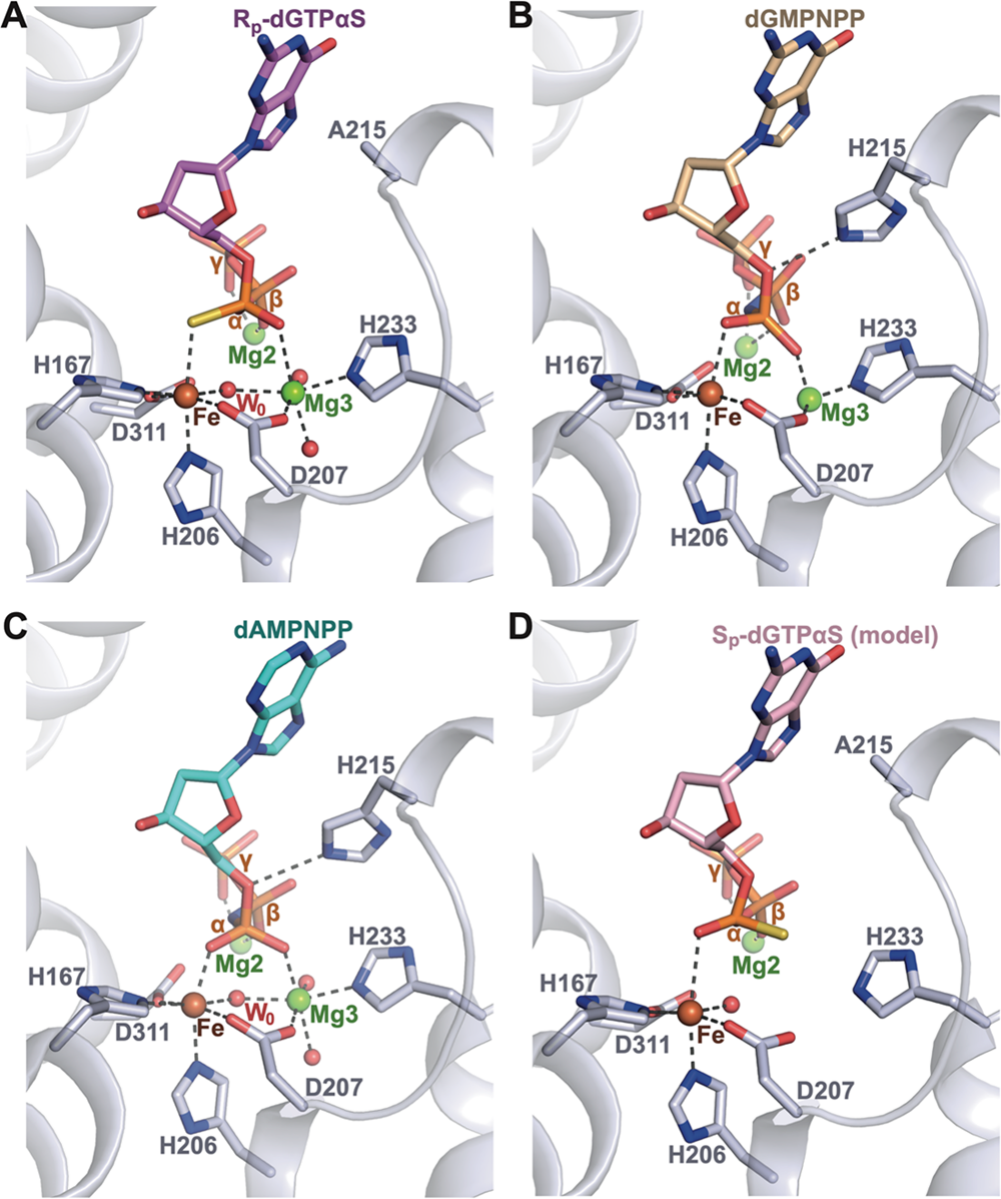
SAMHD1 active site with bound R_p_-dGTPαS. (A) Active site of the cocrystal structure of the H215A-SAMHD1(109–626)-R_p_-dGTPαS complex. (B) Active site of the cocrystal structure of the D137N-SAMHD1(109–626)-XTP-dGMPNPP complex (PDB: 6TXA). (C) Active site of the cocrystal structure of the D137N-SAMHD1(109–626)-XTP-dAMPNPP complex (PDB: 6TX0). (D) Modeling of S_p_-dGTPαS at the active site of H215A-SAMHD1. In each panel, the protein backbone is shown in cartoon representation, and active site Fe and Mg ions and waters are shown as spheres. Bound nucleotides and active site residues are shown in stick representation, colored by atom type, and dashed lines represent the metal ion coordination by HD residues and side chain–nucleotide H-bonding interactions.

Comparison of the configuration of R_p_-dGTPαS bound in the H215A active site with that of dGMPNPP and dAMPNPP inhibitors in wild-type active sites (Figure 7A–C) reveals that the nucleotide coordination, together with the positioning of metal ions and water molecules, is highly conserved. Specifically, the Fe, Mg2, and Mg3 active site metal ions, are equivalently coordinated by side chains from the HD motif residues His167, His206, Asp207, and Asp311, and by the side chain of His233, as well as α, β, and γ-phosphate oxygens and active site water molecules.

In the R_p_-dGTPαS structure, the Fe is coordinated by the α-phosphorothioate sulfur rather than the phosphate oxygen present in a canonical dNTP substrate. Our enzymological data demonstrate that although the S_p_-dNTPαS diastereomers are refractory to hydrolysis they still act as competitive inhibitors of SAMHD1. This indicates that S_p_-dNTPαS diastereomers can still bind the active site, likely through the same electrostatic interactions with the basic side chains of Arg164, Lys312, and Arg366, hydrogen bonds with Gln149 and Tyr315, and π–π stacking with Tyr374 that are observed in the R_p_-dGTPαS structure (Figure 6). Therefore, to assess how the S_p_-diastereomer alters the catalytic competence of the active site, we modeled an S_p_-dGTPαS nucleotide into the R_p_-dGTPαS 2Fo-Fc difference density in our H215A SAMHD1 structure. In the modeled S_p_-dGTPαS structure (Figure 7D), the α-phosphorothioate sulfur and nonbridging oxygen atoms have switched positions. As a result, the α-phosphorothioate nonbridging oxygen coordinates Fe, and the sulfur is now positioned so as to coordinate Mg3 to maintain the octahedral geometry of the coordination sphere.

It is apparent that a sulfur-Mg^2+^ configuration of this kind does not satisfy the pairing-selectivity principle of hard Lewis acid Mg^2+^ cation with a hard Lewis base. Furthermore, analyses of the PDB database reveal that, although coordination of Fe by thiol groups is prevalent in proteins, sulfur coordination of Mg^2+^ does not occur.^69−71^ Therefore, we hypothesize that the loss of coordination between the α-phosphate nonbridging S^2A^ sulfur and Mg3 prevents the formation of a catalytically competent configuration of an S_p_-dNTPαS diastereomer in the active site. One consequence of the absence of this coordination is a diminished electron-withdrawing environment around the α-phosphate, resulting in a reduction of electrophilicity and therefore reactivity of P^α^. In addition, and perhaps more importantly, the hard/soft mismatch between a Mg^2+^ ion and the phosphorothioate thiol moiety could both distort nucleotide binding and result in the loss of Mg3 from the active site.

Regardless of which of these effects dominates, it is unlikely that W0, the hydroxide nucleophile bridged by Fe–Mg3 in the R_p_-dGTPαS structure, could be positioned by an S_p_-dNTPαS nucleotide in line with the P^α^–O^5′^ bond to initiate catalysis. Therefore, overall, our observations support the hypothesis that the hydrolyzable R_p_-dNTPαS nucleotides maintain coordination with the active site Fe and Mg3 through the α-phosphorothioate group and, together with other active site residues, support hydroxide-mediated nucleophilic attack of P^α^ to initiate the P^α^–O^5′^ bond cleavage. By contrast, although S_p_-dNTPαS diastereomers are able to bind at the active site they act as competitive inhibitors, as they cannot maintain the metal and water ion coordination required to support nucleophilic attack on the P^α^.

### Metal Ion Dependencies of dNTP, R_p_-, and S_p_-dNTPαS diastereomers

In order to test our Lewis acid–Lewis base hard/soft mismatch hypothesis, we examined the SAMHD1 metal ion dependency of GTP-stimulated hydrolysis of TTP, R_p_-TTPαS, and S_p_-TTPαS. We first employed a range of divalent metal cations (Mg^2+^, Mn^2+^, Co^2+^, Ni^2+^, Zn^2+^, and Cd^2+^) that constitute hard and softer Lewis acids in the SAMHD1-Ppx1 coupled enzyme assay. However, in control experiments, we found Zn^2+^ and Cd^2+^ did not support triphosphate hydrolysis by Ppx1(Figure S4) and strongly inhibited Ppx1 in the presence of Mg^2+^, so these ions were excluded from further analysis using the coupled enzyme assay. Nevertheless, Zn^2+^ and Cd^2+^ were amenable to ^1^H NMR experiments. These direct assays of TTP hydrolysis showed that Zn^2+^ and Cd^2+^ were also potent inhibitors of SAMHD1 activity, each reducing the SAMHD1 TTP hydrolysis rate >10-fold at 10 μM and >100-fold at 100 μM in the presence of 5 mM Mg^2+^ (Figure S5). These data support previous observations of SAMHD1 inhibition by Zn^2+^^33,72^ and now show Cd^2+^ is similarly effective.

**Figure 8:**
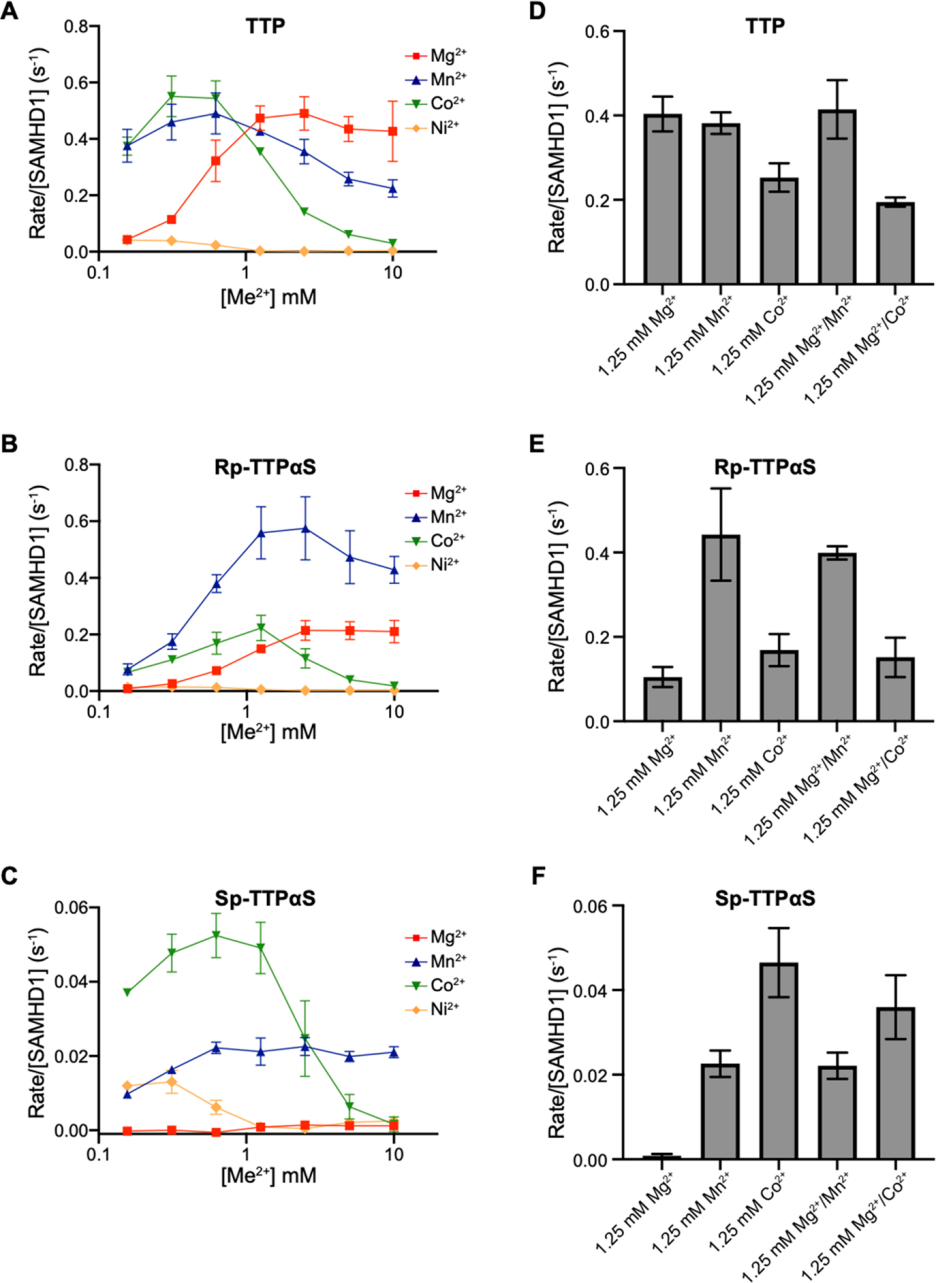
SAMHD1 metal ion dependency of catalysis. (A–C) Dependency of SAMHD1 hydrolysis of (A) 0.5 mM TTP, (B) 0.5 mM R_p_-TTPαS, and (C) 0.5 mM S_p_-TTPαS on different divalent metal ions. The dependence of the enzyme-normalized rate on the concentration of each metal ion is plotted (red) Mg^2+^, (blue) Mn^2+^, (green) Co^2+^, and (yellow) Ni^2+^. (D–E) SAMHD1 enzyme-normalized rate of (D) TTP, (E) R_p_-TTPαS, and (F) S_p_-TTPαS hydrolysis at 1.25 mM divalent metal ion and 1.25 mM each of pairs of divalent metal ions. Error bars are the standard deviation of at least three independent measurements.

Of the remaining divalent metal ions, using the coupled enzyme assay, we found that Ni^2+^ supported very slow hydrolysis of TTP, R_p_-TTPαS, and even S_p_-TTPαS at the lowest Ni^2+^ concentrations employed (0.2–0.4 mM). By contrast, Mg^2+^, Mn^2+^, and Co^2+^ all stimulated hydrolysis to very different degrees depending on the substrate and also with significantly different concentration dependencies (Figure 8A–C). It is apparent that TTP hydrolysis is strongly Mg^2+^ dependent with a maximum stimulation above 1 mM. TTP is also hydrolyzed effectively with Mn^2+^ and Co^2+^, but here the maximum rate is achieved with 0.2–1 mM metal ion, and increased concentration is actually inhibitory to catalysis. This is especially apparent with Co^2+^ (Figure 8A). Hydrolysis of R_p_-TTPαS is also stimulated by Mg^2+^ above 1 mM, but here Mn^2+^ supports faster rates. Similar to the observation with TTP, Co^2+^ also supports hydrolysis at sub-millimolar concentrations but is inhibitory at a higher concentration (Figure 8B). The hydrolysis of S_p_-TTPαS in the presence of Mg^2+^ is below the detection limit, consistent with the notion of the hard/soft mismatch of the Mg^2+^ ion and the phosphorothioate thiol moiety. By contrast, the softer Mn^2+^ and Co^2+^ that can coordinate the phosphorothioate do support hydrolysis of S_p_-TTPαS but also with Co^2+^ being inhibitory at a higher millimolar concentration (Figure 8C). To test if mixtures of metal ions might better support hydrolysis, as there are three divalent metal ion binding sites in each SAMHD1 monomer with potentially different metal ion binding requirements, we determined the rates of hydrolysis with pairs of metal ions at 1.25 mM each. These data (Figure 8D–F and Table 5) largely recapitulate the observations with single metals in that TTP is hydrolyzed effectively by Mg^2+^, and Mn^2+^ and that although Co^2+^ supports hydrolysis it is inhibitory at millimolar concentration even in the presence of Mg^2+^ (Figure 8D). Hydrolysis of R_p_-TTPαS is supported by Mg^2+^, Mn^2+^, and Co^2+^ but is most strongly stimulated by Mn^2+^ that in the background of Mg^2+^ in an ion mixture increases the *k*_cat_ 4-fold rate from 0.1 to 0.4 s^–1^ (Figure 8E and Table 5). Mg^2+^-stimulated hydrolysis of S_p_-TTPαS is not measurable above the limit of detection of the assay (0.002 s^–1^). However, upon addition of Mn^2+^ and Co^2+^ either alone or combined with Mg^2+^, the S_p_-TTPαS hydrolysis rate is increased at least 10-fold by Mn^2+^ and 20-fold by Co^2+^ (Figure 8F and Table 5). Taken together, these data show even though there is a complex relationship between metal ion type, concentration and SAMHD1 substrate, the hydrolysis of S_p_-TTPαS is not supported by the hard Lewis acid Mg^2+^ but can be by softer Mn^2+^ and to a greater extent Co^2+^ ions.

**Table 5:** Metal Ion Dependency of TTP, R_p_-TTPαS, and S_p_-TTPαS Hydrolysis

metal ion^a^	AL 1 activator	substrate	*k*_cat_ (s^–1^)
Mg^2+^	GTP	TTP	0.40 ± 0.04^b^
Mg^2+^	GTP	R_p_-TTPαS	0.10 ± 0.02
Mg^2+^	GTP	S_p_-TTPαS	0.0009^c^ ± 0.0004
Mn^2+^	GTP	TTP	0.38 ± 0.03
Mn^2+^	GTP	R_p_-TTPαS	0.44 ± 0.11
Mn^2+^	GTP	S_p_-TTPαS	0.023 ± 0.003
Mn^2+^ + Mg^2+^	GTP	TTP	0.41 ± 0.07
Mn^2+^ + Mg^2+^	GTP	R_p_-TTPαS	0.40 ± 0.02
Mn^2+^ + Mg^2+^	GTP	S_p_-TTPαS	0.022 ± 0.003
Co^2+^	GTP	TTP	0.25 ± 0.03
Co^2+^	GTP	R_p_-TTPαS	0.17 ± 0.04
Co^2+^	GTP	S_p_-TTPαS	0.047 ± 0.008
Co^2+^ + Mg^2+^	GTP	TTP	0.19 ± 0.01
Co^2+^ + Mg^2+^	GTP	R_p_-TTPαS	0.15 ± 0.05
Co^2+^ + Mg^2+^	GTP	S_p_-TTPαS	0.036 ± 0.008

a 1.25 mM metal ion.

b Error is the SD of three independent measurements.

c value below the reliable limit of detection (0.002 s^–1^).

**Figure 9:**
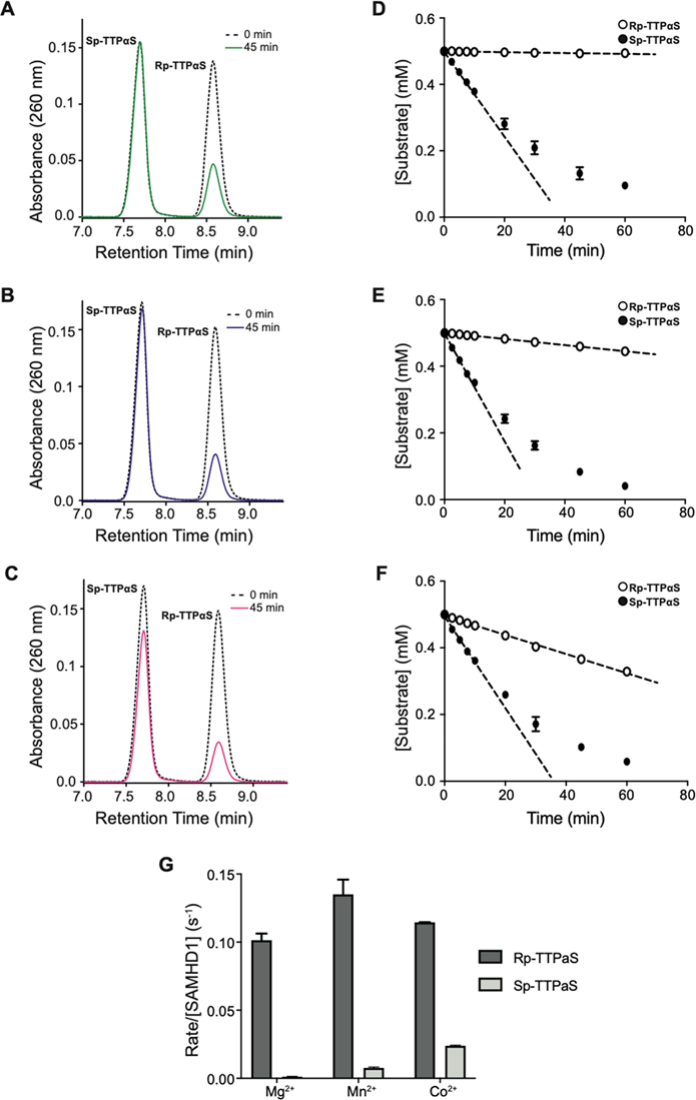
R_p_-TTPαS and S_p_-TTPαS hydrolysis in the presence of Mg^2+^, Mn^2+^, or Co^2+^. (A–C) RP-HPLC traces of hydrolysis reactions containing 2 μM SAMHD1, 0.2 mM GTP, and 0.5 mM each of R_p_-TTPαS and S_p_-TTPαS. Reactions were supplemented with (A) 5 mM Mg^2+^, (B) 1 mM Mn^2+^, and (C) 1 mM Co^2+^. The peaks in the chromatograms are the substrate R_p_-TTPαS and S_p_-TTPαS after a 0 and 45 min reaction. (D–F) Time dependence of SAMHD1 hydrolysis of R_p_-TTPαS and S_p_-TTPαS mixtures at (D) 5 mM Mg^2+^, (E) 1 mM Mn^2+^, and (F) 1 mM Co^2+^. Rates were determined by least-squares fitting of the data in the linear phase of the reactions (dashed lines). (G) Enzyme-normalized rates of reaction for SAMHD1 hydrolysis of R_p_-TTPαS and S_p_-TTPαS mixtures. Data taken from (D–F). Error bars are the standard deviation of least two independent measurements.

To test this notion further and to assess if allosteric binding of R_p_-TTPαS might further enhance S_p_-TTPαS hydrolysis, we examined the divalent metal ion dependency of hydrolysis reactions containing both R_p_-TTPαS and S_p_-TTPαS nucleotides. As ^1^H NMR detection of nucleotide base protons was not possible with the paramagnetic Mn^2+^ and Co^2+^ ions present, to discriminate between hydrolysis of the two substrates in the same reaction, we took advantage of the fact that the diastereomers are separable using ion-pair reverse-phase HPLC. Analysis of a GTP-Mg^2+^-stimulated reaction containing equimolar R_p_-TTPαS and S_p_-TTPαS showed that R_p_-TTPαS is hydrolyzed in the presence of S_p_-TTPαS, while S_p_-TTPαS remains refractory (Figure 9A,D). Nonetheless, the rate of R_p_-TTPαS hydrolysis (Figure 9G) is reduced compared to that of a GTP-Mg^2+^ stimulated reaction of R_p_-TTPαS alone (Figure 8E). Therefore, these data support our conclusions from both NMR and coupled enzyme assays demonstrating that Mg^2+^ cannot support hydrolysis of S_p_-dNTPαS nucleotides and that they are competitive inhibitors of R_p_-dNTPαS nucleotide hydrolysis. In GTP-Mn^2+^- and GTP-Co^2+^-stimulated reactions, some hydrolysis of S_p_-TTPαS along with that of R_p_-TTPαS is observed (Figure 9B,E & C,F) but with no significant increase of the rate (Figure 9G) compared to Mn^2+^- or Co^2+^-stimulated hydrolysis of S_p_-TTPαS alone (Figure 8F). Therefore, these data indicate that while the softer Mn^2+^ and Co^2+^ ions do support SAMHD1 hydrolysis of S_p_-dNTPαS nucleotides the presence of R_p_-dNTPαS nucleotides at allosteric sites does not enhance S_p_-dNTPαS nucleotide hydrolysis further.

## Discussion

Despite the importance of SAMHD1-mediated dNTP regulation of cell proliferation and viral restriction, a proposed catalytic mechanism for dNTP triphosphohydrolysis by SAMHD1 was only recently reported.^44^ Thio-substituted nucleotide analogues are often inhibitory or are poorly hydrolyzed by enzymes, making them useful for structural analysis^73,74^ and have been exploited in a number of mechanistic studies of phospho-hydrolytic enzymes.^65,75,76^ Therefore, in this study, we employed α-thio-substituted R_p_- and S_p_-dNTPαS diastereomers (Figure 1) to probe SAMHD1 catalysis and allostery. Depending on the diastereomer, some SAMHD1 protein-nucleotide interactions are disrupted, while others are maintained, resulting in differences in tetramerization/allosteric activation and in catalysis. Our X-ray crystallographic, enzymological, and biochemical studies using R_p_- and S_p_-dNTPαS diastereomers now provide insight into the specificity of SAMHD1–nucleotide–metal ion interactions at the allosteric and active sites. Moreover, the R_p_-dGTPαS structure provides a model for the enzyme–substrate [ES] complex, while our S_p_-dNTPαS data reveal a new class of SAMHD1 inhibitors that compete for the apo-active site.

### R_p_ and S_p_ Stereoselectivity at the SAMHD1 Allosteric Site

Previous studies have demonstrated the importance of Mg for SAMHD1 activity.^29^ Our present study now highlights the functional importance of these nucleotide–Mg interactions at the allosteric site as demonstrated by the observation that only R_p_-dNTPαS and not S_p_-dNTPαS diastereomers are able to coordinate Mg at AL1 and AL2 to support tetramerization.

At AL1, which is specific for a guanine-based nucleotide, only R_p_-dGTPαS supports tetramerization. Inspection of AL1 in the H215A-SAMHD1(109–626)-R_p_-dGTPαS crystal structure reveals that R_p_-dGTPαS maintains coordination of the AL1-AL2 bridging Mg ion through an α-phosphate oxygen in the same way as a canonical nucleotide. By contrast, with S_p_-dGTPαS the incompatibility of soft Lewis base α-phosphorothioate sulfur and hard Lewis acid Mg disallows this nucleotide-Mg coordination at AL1-AL2 and so is refractory to the subunit packing required for tetramer assembly.

Our biochemical studies reveal less discrimination at AL2 than AL1 but nevertheless do demonstrate that AL2 binding of R_p_-dNTPαS diastereomers stabilizes SAMHD1 tetramerization to a greater extent than S_p_-dNTPαS diastereomers. Here, our structural analysis reveals that R_p_ or S_p_ thio-substitution to the AL2-bound nucleotide has little direct effect on the Mg-coordination. Instead, where a canonical deoxynucleotide or the R_p_-dNTPαS the α-phosphate makes hydrogen bonds with the basic side chains of Lys354 and His376 AL2-interacting residues, the geometry demands that in an Sp-dNTPαS the S_p_-phosphorothioate is required to make these interactions. Given the reduced electronegativity of sulfur relative to oxygen and that it is a very poor hydrogen bond acceptor,^77^ a loss of this hydrogen bonding likely explains the reduced capacity of S_p_-dNTPαS deoxynucleotides to support SAMHD1 tetramerization through binding at AL2. Therefore, taken together, it is apparent that both allosteric sites discriminate R_p_ over S_p_, but the selection is mediated in different ways. At AL1, it is through the loss of a direct interaction with the Mg ion and at AL2 it is through the lack of capacity for a S_p_-phosphorothioate to make hydrogen bonding interactions with the key residues that support tetramerization upon nucleotide binding.

### R_p_-dNTPαS Hydrolysis and S_p_-dNTPαS Inhibition of SAMHD1

Our enzymological and biochemical data clearly show that the structural differences arising from the stereochemistry of R_p_- and S_p_-dNTPαS analogues have significant effects on SAMHD1 activity. R_p_-dNTPαS nucleotides are substrates of SAMHD1 with catalytic constants comparable with those of canonical nucleotides. In contrast, S_p_-dNTPαS nucleotides are inhibitors of SAMHD1 triphosphohydrolase activity, likely through binding competitively at the active site.

To understand these observed differences, we employed a SAMHD1 mutant, H215A, which retains nucleotide binding but is catalytically deficient^44^ to determine the structure of SAMHD1 in complex with a substrate R_p_-dGTPαS at the active site (Figure 6). The use of this mutant in combination with substrate R_p_-dGTPαS has now enabled us to visualize a substrate precatalysis in the SAMHD1 active site for the first time and so provides an excellent structural tool for studying other SAMHD1 substrates, such as canonical dNTPs and nucleotide-based anticancer and antiviral drugs. These data demonstrate how a substrate R_p_-dNTPαS is positioned in the SAMHD1 active site. Unlike in previous structures, the H215A-SAMHD1(109–626)-R_p_-dGTPαS–Mg complex reveals how the substrate R_p_-dGTPαS is poised for nucleophilic attack by an Fe–Mg-bridged water species, W0, likely a hydroxide ion (Figures 6 and 7 and Supplementary Figure S3). Moreover, the substrate R_p_-dGTPαS binding conformation is highly similar to that of a dNMPNPP inhibitor, which, we previously proposed, mimics the precatalytic state.^44^ This is despite the substitution of an α-phosphate nonbridging oxygen with the phosphorothioate in R_p_-dGTPαS, which nevertheless still supports Fe coordination and nucleotide hydrolysis.

Modeling of the S_p_-dNTPαS diastereomers at the SAMHD1 active site shows there is a similar incompatibility between the S_p_-α-phosphorothioate and Mg3 as with the Mg1 and S_p_-dGTPαS in the allosteric site. Here the S_p_-thio moiety would have to approach Mg3 in the active site, but due to the sulfur/magnesium mismatch, this likely distorts nucleotide binding in the catalytic site to the extent that the attacking hydroxide nucleophile, W0, and substrate nucleotide are not aligned for catalysis. We have previously demonstrated the importance of Mg3 by alanine substitution of the Mg3-coordinating residue His233 that resulted in a 300-fold reduced *k*_cat_ for GTP-activated dATP hydrolysis.^44^ Thus, our observation here that S_p_-dNTPαS diastereomers bind in the active site, are competitive inhibitors and are not hydrolyzed by SAMHD1 further supports the notion that the Fe-proximal Mg is crucial for catalysis.

The idea of the hard–soft mismatch between the α-phosphorothioate of S_p_-dNTPαS diastereomers with Mg3 is further supported by our metal ion dependency experiments. We employed a range of hard to soft metal ions to ascertain whether hydrolysis of S_p_-dNTPαS diastereomers could be rescued by employing softer metal ions that support interaction with the S_p_ α-phosphorothioate. These data showed convincingly that, while hard Mg^2+^ did not support hydrolysis of S_p_-dNTPαS, the S_p_ diastereomer was hydrolyzed in the presence of the softer Mn^2+^ and Co^2+^ metal ions.

### Mechanisms of Inhibition

**Figure 10:**
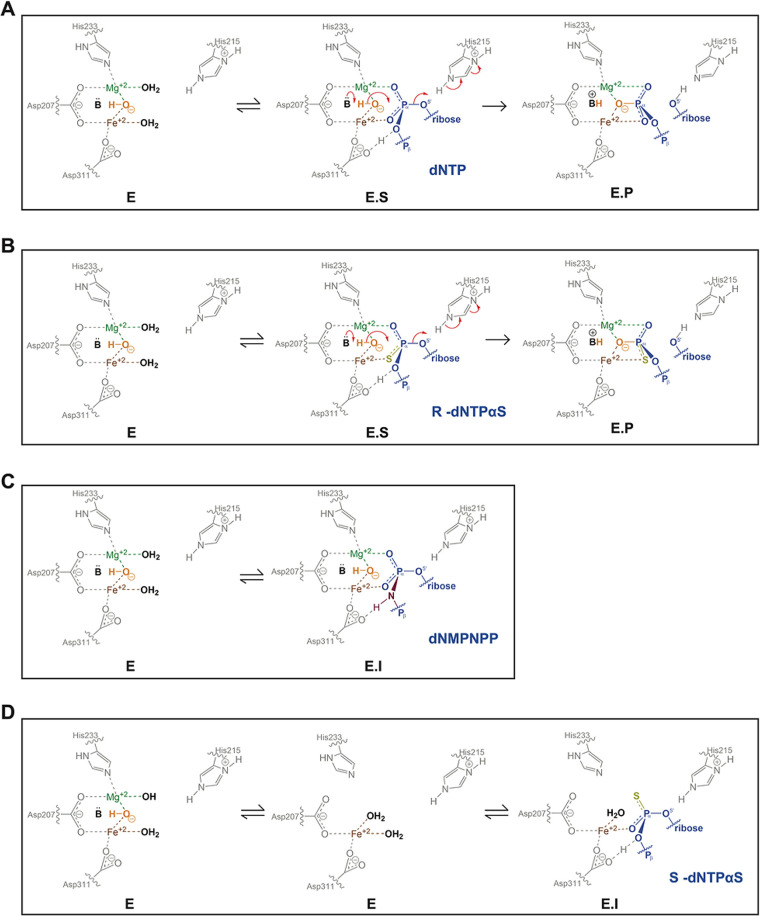
SAMHD1 catalytic mechanism and inhibition. (A and B) Schematic of the chemical mechanism of SAMHD1 hydrolysis of canonical dNTPs and R_p_-dGTPαS nucleotides. In the apo state [**E**], the W0 water molecule (orange) is coordinated between the HD motif bound Fe ion and by Mg3; further water molecules and protein side chains take up the remaining coordination positions on the metal ions. On substrate binding, the enzyme–substrate complex (**E·S**) is formed, and the P^α^ oxygens of canonical dNTPs or the α-phosphorothioate and α-phosphate in the R_p_-dNTPαS nucleotide replace the water molecules to coordinate the active site Fe and Mg3 respectively and also position the W0 nucleophile in line with the electron-deficient α-phosphate. The reaction proceeds by adduction of the W0 nucleophile to the α-phosphate. The resulting accumulating negative charge is relieved by protonation of the leaving nucleoside 5′ oxygen by His215 to form the enzyme product complex [**E·P**]. (C) dNMPNPP inhibition. dNMPNPP nucleotides can still engage the active site Fe and Mg3 respectively and also position the W0 nucleophile. However, the additional hydrogen bond between the Asp311^Oδ^ and the H^imido^ of the dNMPNPP forms a stable inhibitor complex [**E·I**] that prevents formation of the transition state and bond inversion. (D) S_p_-dNTPαS inhibition. S_p_-dNTPαS nucleotides compete for active site binding through interactions with Fe and surrounding coordinating side chains, but they are unable to coordinate Mg3. Instead, they form a transient **E·I** complex that cannot position the hydroxyl nucleophile and support catalysis.

Given the notion that S_p_-dNTPαS diastereomers bind at the active site and act as competitive inhibitors but cannot engage with the catalytic metal ions to enable the catalytic geometry means that they represent a different class of SAMHD1 inhibitor from those reported previously.^41,44,78^Figure 10 shows a comparison of the reaction mechanism schemes for canonical dNTP and R_p_-dNTPαS and also for inhibition by dNMPNPP and S_p_-dNTPαS nucleotides. In these proposed reaction mechanisms, dNTP and R_p_-dNTPαS (Figure 10A,B) follow the same profile with the α-phosphates in the canonical nucleotide or α-phosphorothioate and α-phosphate in the R_p_-dNTPαS nucleotide first coordinating the active site Fe and Mg3 respectively. The reaction then proceeds through adduction of the hydroxyl nucleophile at the α-phosphate of the ES complex to a trigonal bipyramidal transition state. Inversion of P^α^ and breakage of the P^α^–O^5′^ bond, catalyzed by His215 acting as a general acid, then results in incorporation of W0 into the newly formed triphosphate product and concomitant release of the 2′-deoxynucleoside. The proposed mechanism of inhibition by dNMPNPP nucleotides (Figure 10C) is through increased stability of an EI complex by a Asp311^Oδ^ and H^imido^ hydrogen bond. So, although the EI complex mimics the ES complex with all metal ions in place as well as the catalytic hydroxide molecule, the increased stability of the EI complex prevents formation of the transition state and bond inversion. For the S_p_-dNTPαS nucleotides, we now propose an alternative mechanism of inhibition (Figure 10D). Here, although S_p_-dNTPαS nucleotides can bind in the active site through interactions with Fe as well as with surrounding active site side chains, they may adopt a configuration that is unable to coordinate the Mg3 metal ion and hydroxyl nucleophile. Accordingly, they represent a nonproductive EI complex that cannot assemble further into an ES complex and support catalysis.

Our results with SAMHD1 reiterate many previous observations regarding the exquisite stereoselectivity of enzymes. They show on one hand how the analysis of the differential effects of diastereomer pairs of substrate, activator, and inhibitor molecules is a powerful tool to inform on the enzyme mechanism and protein structure. Using this approach, we have uncovered two modes of competitive inhibition of SAMHD1 by nucleotide-based compounds at the active site. Type-I is exemplified by dNMPNPP nucleotides that inhibit through competition with the ES complex. Type-II, exemplified by the S_p_-dNTPαS nucleotides, represents a new mode of inhibition that works through competition with the initial binding of substrate nucleotides to form a transient EI complex with a conformation that does not engage the hydroxyl nucleophile. Given the need to modulate SAMHD1 activity to better understand its cellular functions, both of these modes of inhibition now provide starting points for the discovery of tool compounds that can be used to understand SAMHD1 function in HIV-1 restriction, DNA repair, and innate immune sensing.

## Supporting Information Available

Table of X-ray data collection and refinement statistics, table of primers used for cloning and mutagenesis, and figures describing the crystallographic AU, electron density for nucleotides bound in the allosteric and active sites, metal dependancy of Ppx1 triphosphate hydrolysis and zinc.cadmium inhibition of SAMHD1(PDF)

## Accession Codes

The atomic coordinates and structure factors of the H215A-SAMHD1(109-626)-R_p_-GTPαS complex have been deposited in the Protein Data Bank under accession number 7A5Y.
