# Supplementary material for: Probing the Catalytic Mechanism and Inhibition of SAMHD1 Using the Differential Properties of Rp- and Sp-dNTPαS Diastereomers
Source: Biochemistry. 2021 May 14;60(21):1682–98. doi: 10.1021/acs.biochem.0c00944 (PMC8173608; doi:10.1021/acs.biochem.0c00944)
Supplement: Supplementary file 1 — bi0c00944_si_001.pdf [file bi0c00944_si_001.pdf]

Supporting Information

Probing the catalytic mechanism and inhibition of SAMHD1 using the differential properties of R_p- and S_p-dNTPαS diastereomers.

Elizabeth R. Morris[†1], Simone Kunzelmann[2†], Sarah J. Caswell[1#], Andrew G. Purkiss[2], Geoff Kelly[3] and Ian A. Taylor[1*]

[1]Macromolecular Structure Laboratory, [2]Structural Biology Science Technology Platform, [3]The Medical Research Council Biomedical NMR Centre, The Francis Crick Institute, 1 Midland Road, London NW1 1AT, UK.

[#]Present address: AstraZeneca, Aaron Klug Building, Granta Park, Cambridge CB21 6GH.

[†]Equal contributing authors

*Corresponding author Ian A Taylor

E-mail: ian.taylor@crick.ac.uk

Telephone: +44 (0) 20 3796 2288

Table S1: X-ray data collection and structure refinement statistics.

Table S2. Primers for SAMHD1 cloning and mutagenesis.

Figure S1. H215A-SAMHD1(109-626)-R_p-dGTPαS complex, crystal structure asymmetric unit.

Figure S2. Electron density for R_p-dGTPαS bound at the allosteric site.

Figure S3. Electron density for R_p-dGTPαS bound at the H215A active site.

Figure S4. Metal dependency of Ppx1-hydrolysis of triphosphate.

Figure S5 Inhibition of SAMHD1 TTP hydrolysis by Zn^{2+} and Cd^{2+}.

Table S1. X-ray data collection and structure refinement statistics

	H215A-SAMHD1 Mg, R_p-dGTPαS	H215A-SAMHD1 Mg, R_p-dGTPαS (STARANISO)
**Data collection**		
Space group	$P\ 2_1\ 2_1\ 2_1$	$P\ 2_1\ 2_1\ 2_1$
Cell dimensions		
a, b, c (Å)	80.4, 181.6, 286.9	80.2, 181.3, 286.3
α, β, γ	90, 90, 90	90, 90, 90
Wavelength (Å)	0.9795	0.9795
Resolution (Å)	66.74(2.54)-2.50	153.15(2.45)-2.29
Anisotropic diff. limits a*, b*, c* (Å)	-	2.69, 2.37, 2.29
Unique reflections	146,247 (7,156)	145,900 (7,295)
R_{meas} (%)	23.8 (117)	28.7 (167)
R_{pim} (%)	9.1 (44.1)	11.0 (64.6)
$CC_{1/2}$	0.989 (0.673)	0.989 (0.546)
I/δ(I)	5.4 (0.9)	6.3 (1.6)
Completeness		
Spherical (%)	100.0 (99.6)	77.2 (20.5)
Ellipsoidal (%)	-	92.6 (57.6)
Multiplicity	6.7 (6.9)	6.8 (6.5)
**Refinement**		
Resolution (Å)		153.15-2.29
R_{work}/R_{free}/Test set (%)		21.1/24.0/5.0
No. monomers/A.S.U.		8
No. atoms		
Protein		30,465
Nucleotide		248
Ligand Fe		8
Ligand Mg		24
Water		343
B-factors (Å)		
Wilson		30.7
Protein		39.7
Nucleotide		24.2
Ligand Fe		26.5
Ligand Mg		35.3
Water		22.7
Average		38.6
R.m.s. deviations		
Bond lengths (Å)		0.0178
Bond angles (°)		1.9148
Chiral volumes (Å^3)		0.1102
Ramachandran (%)		
Favoured		97.1
Allowed		2.8
Outliers		0.1
PDB code		7A5Y

†Values in parentheses refer to the highest resolution shell

Table S2. Primers for SAMHD1 cloning and mutagenesis

Construct		Primers (5' - 3')*#
M1-M626	FWD	CAGGGACCCGGTATGCAGCGAGCCGATTCCGAGCAG
(full length)	REV	GGCACCAGAGCGTTACATTGGGTCATCTTTAAAAAGCTG
Q109-M626	FWD	GGCCCCGGGCAAATCCACGTTGATACAATG
	REV	GGCGCGGCCGCTCATCACATTGGGTCATCTTTAAAAAGCTGG
H215A	FWD	GTCATGGGCCATTTTCTGCCATGTTTGATGGACGATTTATTC
	REV	GAATAAATCGTCCATCAAACATGGCAGAAAATGGCCCATGAC

*LIC or restriction sites used for cloning are underlined; #Mutagenized codons are highlighted and underlined.

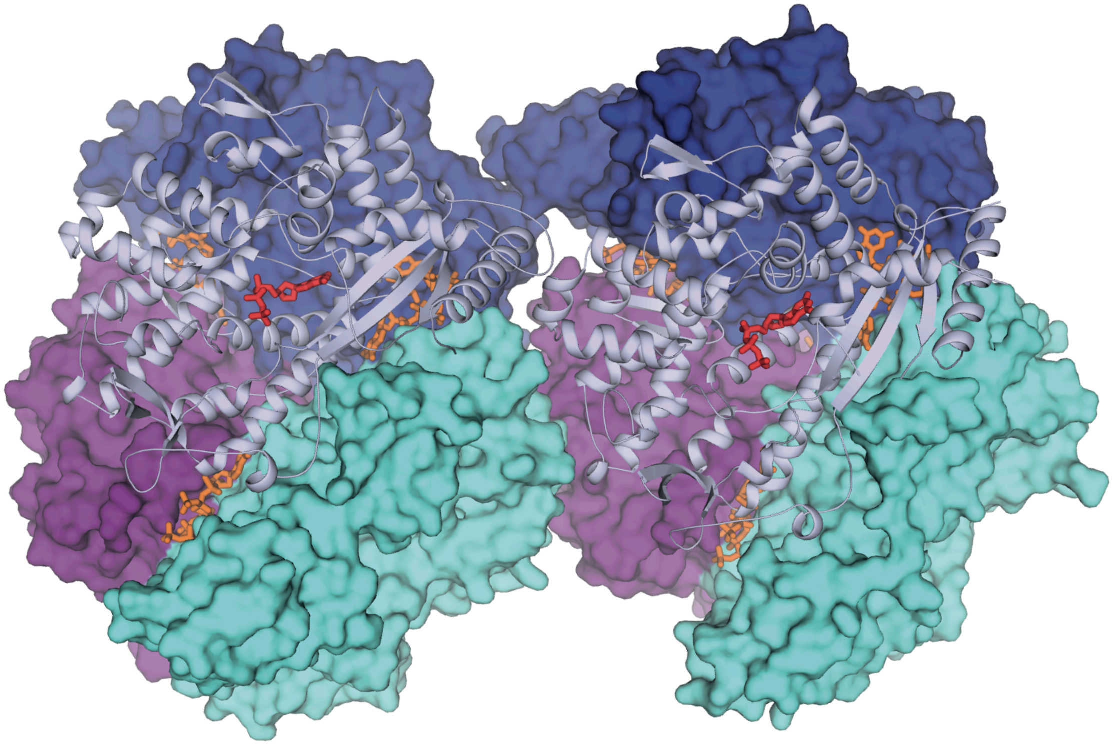

Figure S1. H215A-SAMHD1(109-626)-R_p-dGTPαS complex, crystal structure asymmetric unit. The Crystal structure of H215A-SAMHD1(109-626)-R_p-dGTPαS complex contains two homo-tetramers. In each tetramer the monomers are shown in surface representation with a single monomer shown in cartoon representation. Monomers are colored cyan, dark purple, dark blue and blue/white, respectively. Nucleotides bound at the active and allosteric site in the cartoon monomer are shown in stick representation and colored red and orange, respectively.

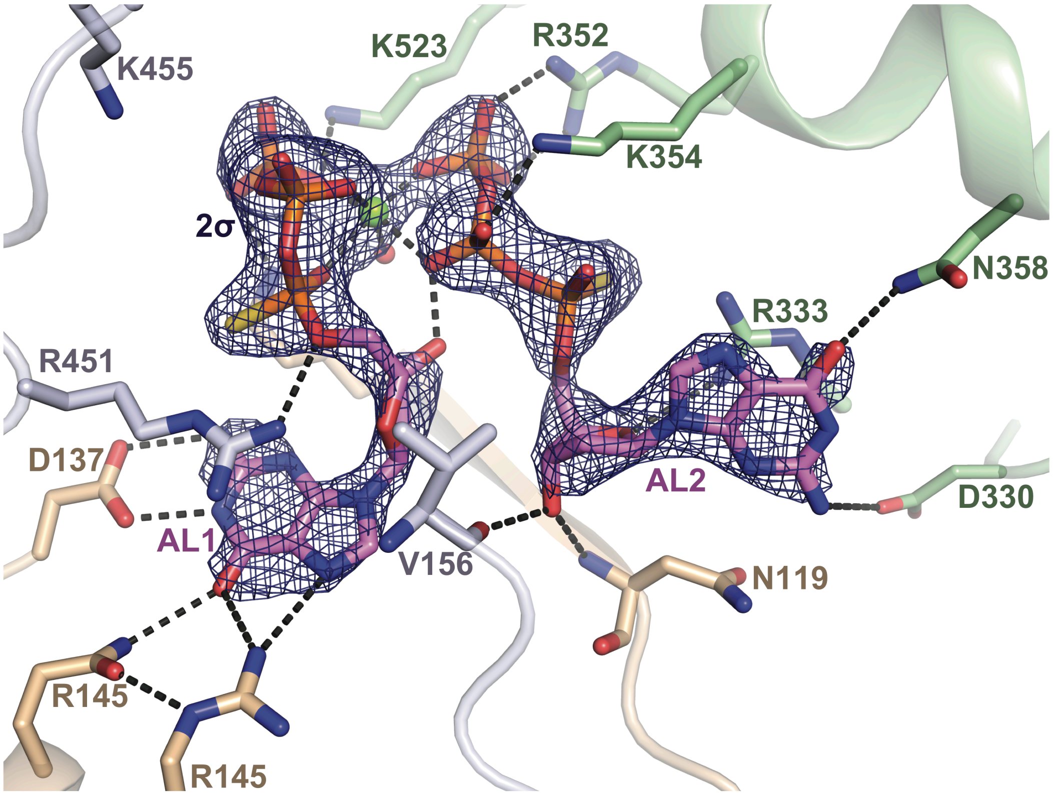

Allosteric site

Figure S2. Electron density for R_p-dGTPαS bound at the allosteric site. The protein backbone is shown in cartoon representation, with monomers colored wheat, blue-white and pale green. The 2Fo-Fc simulated annealing composite omit map for R_p-dGTPαS nucleotides in AL1 and AL2 and AL1-AL2-bridging Mg is shown as a blue mesh, contoured at 2.0σ. Nucleotides and residues surrounding AL1 and AL2 are shown in stick representation. The Mg ion is shown as a green sphere. Hydrogen and co-ordinate bonds are displayed as dashed grey lines.

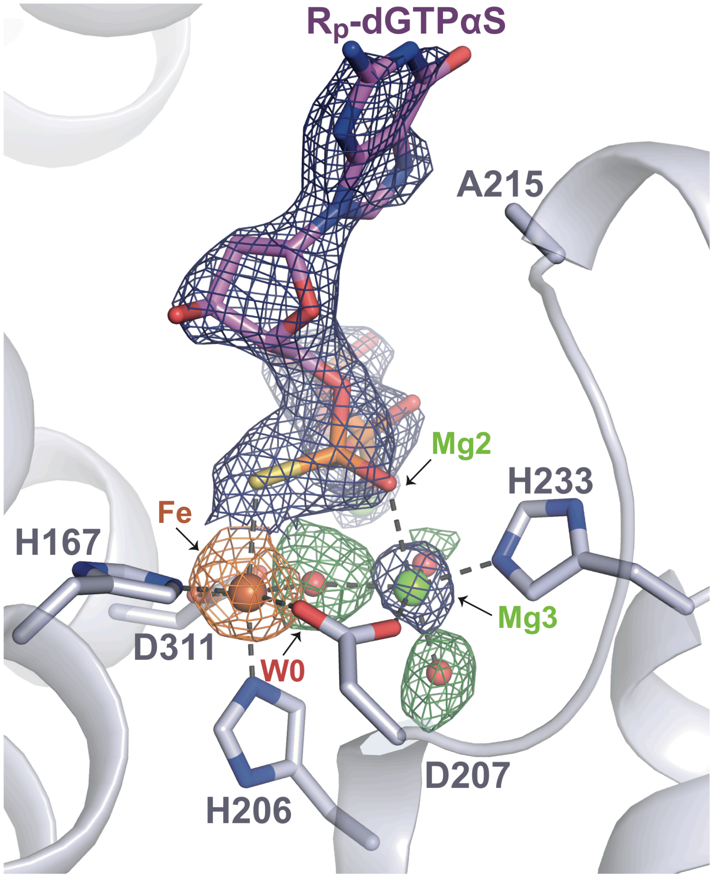

Figure S3. Electron density for R_p-dGTPαS bound at the H215A active site. The SAMHD1 protein backbone is shown in cartoon representation, colored blue-white, and the active site-bound R_p-dGTPαS nucleotide is shown in stick representation. Fe and Mg ions are represented as brown and green spheres, respectively. The simulated-annealing composite omit 2Fo – Fc electron density (blue mesh) for the active site bound R_p-dGTPαS nucleotide and two Mg ions is shown contoured at 2.2σ. The Fo-Fc difference density prior to atom placement is shown as a mesh for Fe (5σ, brown) and W_0 and Mg-coordinated waters (2.5σ, green). Hydrogen and co-ordinate bonds are displayed as dashed grey lines.

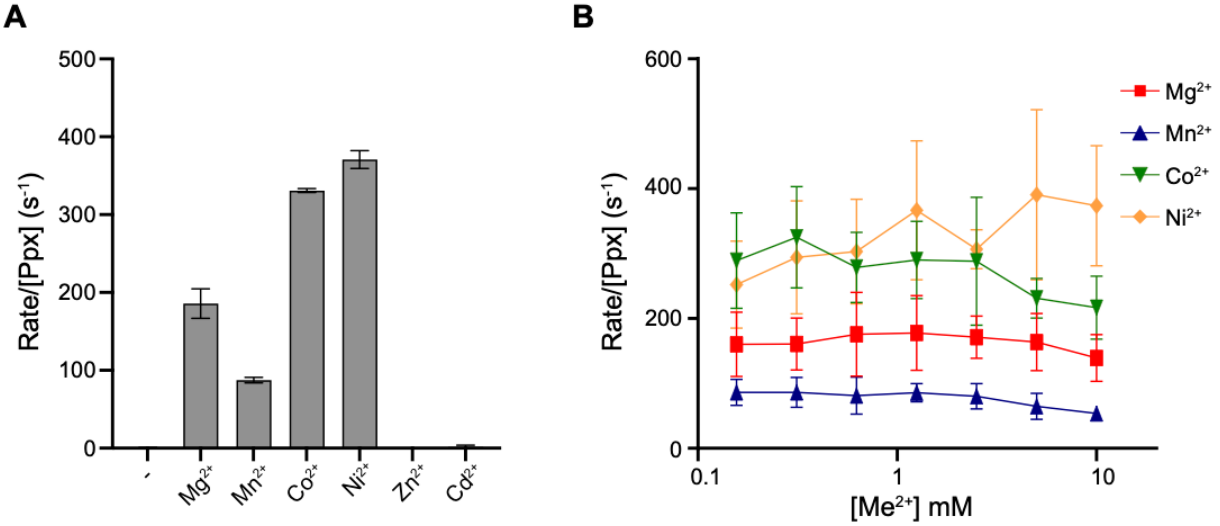

Figure S4. Metal dependency of Ppx1-hydrolysis of triphosphate. (A**)** Enzyme normalized rates of triphosphate hydrolysis by Ppx1 (0.05 nM) with added 5 mM Mg^{2+}, Mn^{2+}, Co^{2+}, Ni^{2+}, Zn^{2+} and Cd^{2+} divalent metal ions (-, no metal ion), Zn^{2+} and Cd^{2+} do not support hydrolysis. (B) The dependence of the enzyme-normalized rate on the concentration of each metal ion (0.1- 10mM) that support Ppx1 catalysis are plotted, (red) Mg^{2+}, (blue) Mn^{2+}, (green) Co^{2+} and (yellow) Ni^{2+}. Error bars in both panels are standard deviation from at least two independent measurements.

A

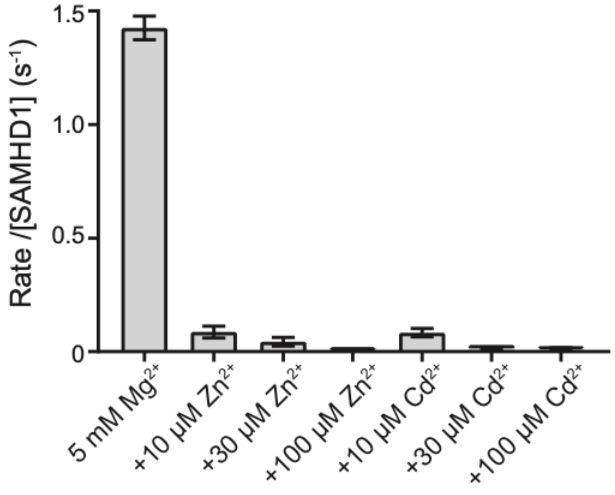

B

**Me^{2+}**	**k_{cat} (s^{-1})**	***f***
5 mM Mg^{2+}	1.43±0.05	-
+10 μM Zn^{2+}	0.087±0.026	16
+30 μM Zn^{2+}	0.043±0.019	33
+100 μM Zn^{2+}	0.0063±0.0020	227
+10 μM Cd^{2+}	0.084±0.018	17
+30 μM Cd^{2+}	0.014±0.005	102
+100 μM Cd^{2+}	0.0099±0.0069	144

Figure S5 Inhibition of SAMHD1 TTP hydrolysis by Zn^{2+} and Cd^{2+}. (A) Bar chart of enzyme-normalized rates derived from ^{1}H NMR analysis of GTP-activated SAMHD1 hydrolysis of TTP with 5 mM Mg^{2+} and increasing concentration of Zn^{2+} or Cd^{2+} (10-100 μM). Error bars are SEM from at least three independent measurements. (B) Enzyme-normalized rates of SAMHD1 TTP hydrolysis. The data is taken from (A), *f* is the fold reduction observed upon addition of Zn^{2+} or Cd^{2+} to hydrolysis reactions.
